# A narrative review of outcome measures used in drug and alcohol inpatient withdrawal treatment research

**DOI:** 10.1111/dar.13591

**Published:** 2023-01-12

**Authors:** Jing Wang, Frank P. Deane, Peter J. Kelly, Laura Robinson

**Affiliations:** ^1^ School of Psychology University of Wollongong Wollongong Australia; ^2^ Illawarra Health and Medical Research Institute Wollongong Australia

**Keywords:** drug and alcohol use, inpatient withdrawal treatment, outcome assessment, withdrawal treatment outcomes

## Abstract

**Issues:**

Assessing drug and alcohol inpatient withdrawal treatment programs is important, as these represent a first step of treatment among people with alcohol and drug problems. However, there are many ways of measuring outcomes making it difficult for service providers to decide which domains and methods to use. This narrative review aims to clarify frequencies of the domains and methods used to assess withdrawal treatment outcomes.

**Approach:**

We reviewed published studies that examined outcomes of inpatient drug and alcohol withdrawal treatment. The types of outcome measures used and the frequency of use were summarised.

**Key Findings:**

The review showed that assessment of withdrawal treatment outcomes goes beyond traditional abstinence measures. Outcomes mainly focus on biological and psychological outcomes, with social outcomes rarely measured. Even within outcome domains (e.g., cravings), there were many assessment methods.

**Implications:**

The review provides service providers with an outline of common outcome domains and measures. Given the importance of social functioning to recovery from alcohol and drug problems, greater emphasis on such measures is desirable. Future research could develop greater consensus on outcome measures for use in withdrawal management services to facilitate clarity around factors associated with treatment success.

**Conclusion:**

Outcome assessment in withdrawal treatment goes beyond abstinence to include holistic measurement of biological, psychological and some social outcomes; but more work needs to be done to cohere the different assessment methods and broaden the scope to include social functioning.

## 1 INTRODUCTION

Mental health and substance use disorders were the fifth largest contributors to disability‐adjusted life years (DALY) globally in 2010 and were responsible for 7.4% (or 183.9 million) of DALYs [1]. Together, alcohol use disorders and drug use disorders account for 20.5% of this DALY burden, second only to depressive disorders [1]. In Australia, drug and alcohol use disorders accounted for 24% of the DALY burden in 2015 [2]. From 2020 to 2021, 139,300 clients aged at least 10 years received treatment for substance abuse in Australia, totalling 239,000 treatment episodes [3].

Given that withdrawal treatment often serves as a patient's first point of contact with formal treatment, it is imperative that the effectiveness of such services is understood. However, a challenge has been the wide range of outcome measures used in assessing withdrawal treatment outcome, and a lack of clarity regarding the relative strengths and weaknesses of these measures. Existing studies have used the following outcome assessments: (i) ‘safe’ withdrawal described as the prevention of severe withdrawal sequelae and minimisation of distress associated with withdrawal [4, 5, 6, 7]; (ii) successful completion of withdrawal treatment [4, 8, 9]; (iii) abstinence during withdrawal treatment [9]; (iv) abstinence rates at follow‐up (i.e., after discharge; [8, 10, 11]); (v) engagement in continuing care after discharge [8, 11, 12, 13]; (vi) withdrawal treatment satisfaction [5, 14]; and (vii) miscellaneous aspects of client functioning (e.g., rates of homelessness and employment; [8, 15, 16]).

Considering the numerous and varying measures of withdrawal treatment success across the literature, the current narrative review sought to clarify the frequencies of the different types of outcome assessments used in inpatient withdrawal treatment services.

## 2 METHOD

### 2.1 Narrative review

A narrative review was utilised due to the diversity of the topic and the wide range of methods used in the relevant studies. Additionally, the aim of this paper, investigating the frequency of outcome assessment types, is quite broad. As the research in this area does not always have an agreed‐upon gold standard methodology like double‐blind‐randomised‐controlled‐trials, narrative literature reviews are often viewed as appropriate and capable of advancing theoretical and conceptual understanding [17, 18]. Thus, a narrative versus other forms of systematic review was chosen.

### 2.2 Search strategy

To identify studies for review, we performed a Boolean search in the Medline, Scopus, PsycInfo, PsycArticles, PsycBooks and Psychiatry Online databases, using the search terms in abstract: (‘inpatient’ or ‘residential’) AND (‘detoxification’ or ‘withdrawal management’) OR in title: ‘inpatient’ AND ‘detoxification’. The use of a limited number of databases was due to the large number of results produced and a significant overlap of papers between databases. This study was registered on PROSPERO (CRD42021088576).

The search, conducted on 2 November 2021, was limited to studies reported in English language journal articles. We excluded preprints, commentaries, case studies, errata, study protocols and reviews/meta‐analyses. Trade publications and grey literature were not included since we focused on peer‐reviewed studies because these are more likely to include designs of sufficient rigour and that the results are most likely to be used to inform subsequent recommendations for assessment and treatment processes in services [19]. Additionally, funders and advocacy groups strongly endorse use of ‘evidence‐based approaches’ [20] that are more likely to be derived from peer‐reviewed publications. To focus on recent developments in the field, the search was limited to articles published from 2012. We wanted to look at a more recent and updated conceptualisation of drug and alcohol recovery, hence we used articles that came out after the Substance Abuse and Mental Health Services Administration (SAMHSA) recovery model was published. This would in theory provide a more holistic view of recovery including biological, psychological and social functioning [21]. Previously, recovery was defined mostly by abstinence, but recovery conceptualisations have now been expanded to include multiple facets such as, stable housing, health and sense of purpose [21].

### 2.3 Inclusion and exclusion criteria

**FIGURE 1:**
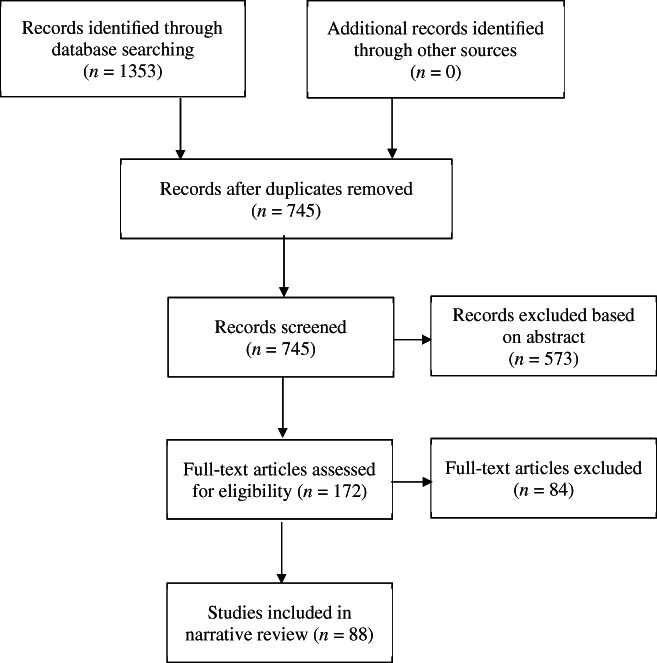
Flowchart of the literature review process

The literature review process is shown in Figure 1. The search produced 1353 articles; after duplicates were removed, 745 remained. Article abstracts were screened for eligibility by one reviewer using the following criteria: (i) mentioned residential or inpatient withdrawal treatment; (ii) mentioned at least one outcome measure related to residential or inpatient withdrawal treatment; (iii) implemented at least one intervention (we chose to focus on intervention studies because they specify the primary outcomes more frequently. This is considered important because it gives some indications of which outcomes are more important by researchers); and (iv) outcomes were measured in relation to inpatient or residential withdrawal treatment only and not a mixture of inpatient or residential withdrawal treatment and other kinds of treatment (e.g., outpatient treatment, rehabilitation). This screening resulted in 573 articles being removed.

The remaining 172 articles were shortlisted for full text screening. Articles were then assessed for inclusion into the narrative synthesis if they met the specified criteria and included a sufficient description of the withdrawal treatment intervention for data extraction. Full texts were independently assessed for eligibility by the first author and a second reviewer. There was a high degree of agreement between the two reviewers (*k* = 0.85, *p* < 0.001) and any discrepancies were resolved through discussion. After the full‐text review, 88 studies were shortlisted for inclusion in the narrative review. Of the 84 studies excluded after the full text review, 66 were due to not describing the withdrawal treatment procedure or not providing a sufficient description for narrative synthesis data and 18 were because they mixed inpatient or residential withdrawal treatment with other kinds of treatment, such as outpatient treatment or rehabilitation.

### 2.4 Data extraction

Data extracted included brief sample characteristics, withdrawal treatment program components, outcome measures used, main findings and percentages of participants who completed withdrawal treatment and attended follow‐up treatment, if applicable. A summary of each of the 88 eligible studies is presented in three different tables, available as Supporting Information: Table S1 (alcohol users), Table S2 (opioid users) and Table S3 (other drug users), where the main findings column aims to detail how outcomes are reported/used.

## 3 RESULTS

### 3.1 Description of eligible studies

Twenty‐two studies were conducted in the USA, 13 in Germany, 6 in the Netherlands, 5 in Australia, 4 each in Brazil, Belgium, India and Iran, 3 each in Canada, Italy and Spain, 2 each in Austria, Sweden and the United Kingdom, and 1 each in 11 other countries.

Included studies mainly examined people with alcohol use disorders (30 studies), opioid use disorder (22 studies) and mixed substance use disorders (20 studies). To a lesser extent, cocaine dependence, cannabis dependence and heroin dependence were examined by eight, five and three studies, respectively.

Males comprised the majority of the sample in 78 (88.6%) of the 88 studies. Mean ages ranged from 11.14 [22] to 54.1 years [23] in the 82 studies that reported mean age. Sample sizes ranged from 11 to 72,205.

### 3.2 Description of program components

Twenty‐six studies (29%) used a combination of pharmacological and psychological interventions, and 61 studies (69%) used only pharmacological interventions. One study (1%) used only psychological interventions.

### 3.3 Description of outcome measures

#### 3.3.1 Withdrawal treatment completion rate

Overall, 28 studies (31.8%) reported the withdrawal treatment completion rate. In order to estimate a mean completion rate across studies, when a range of completion rates was reported, we used the midpoint for calculation (e.g., range 90% to 100% then 95% used). The mean completion rate across studies was 74.9%.

Completion rates were defined by either: clinician judgement (13 studies) or completing a specified length of treatment (14 studies). One study [24] defined completion as either completing a specified treatment or withdrawal management according to clinician judgement.

In order to determine completion rates according to drug problem, we only considered drug problem types with at least three studies. For example, we did not analyse completion rates for gamma hydroxybutyrate dependence as only one study was identified [25].

Of the 30 studies concerning alcohol dependence, 5 reported completion rates, ranging from 71.3% to 98.3% (*M* = 86.2%). Of the 21 studies concerning opioid dependence, 11 reported completion rates, ranging from 56.0% to 100% (*M* = 68.8%). Of the 16 studies concerning mixed substance dependence, 5 reported completion rates, ranging from 44.0% to 99% (*M* = 73.2%). Of the nine studies concerning cocaine dependence, only one reported a completion rate which was 79.5%. Of the five studies concerning cannabis dependence, four reported completion rates, ranging from 45.5% to 88.6% (*M* = 77.4%). Of the three studies concerning heroin dependence, none reported completion rates.

#### 3.3.2 Treatment continuity

Follow‐up treatment attendance rates were reported in seven studies, ranging from 9.7% to 84.4%. Follow‐up treatment was defined as attending treatment after discharge from withdrawal treatment, based on reviewing official records at the follow‐up treatment facility (three studies [11, 26, 27]) or via self‐report (two studies [12, 28]). Two studies [29, 30] did not specify how they obtained follow‐up treatment attendance rate data.

#### 3.3.3 Other outcome measures

##### Withdrawal symptoms

Thirty‐two studies reported outcomes related to withdrawal symptoms. The most frequent measure, utilised in nine studies, was the self‐reported Subjective Opioid Withdrawal Scale [23, 31, 32, 33, 34, 35, 36, 37, 38]. Other outcome measures included the amount of ancillary or rescue medication used (five studies [35, 39, 40, 41, 42]), self‐report via the Clinical Institute Withdrawal Assessment for Alcohol, Revised Version (CIWA‐Ar) (four studies [39, 43, 44, 45]) or Clinical Institute Withdrawal Assessment for Alcohol (CIWA) (one study [46]). Clinician report via the Objective Opiate Withdrawal Scale was used in four studies [33, 35, 42, 47] and self‐report via the Cocaine Selective Severity Assessment in four studies [48, 49, 50, 51]. The clinician‐rated Clinical Opiate Withdrawal Scale was used in three studies [31, 32, 52] as was the clinician‐rated Clinical Global Impression Scale [42, 53, 54]. The self‐report Cannabis Withdrawal Scale [55, 56], the Adjective Rating Scale for Withdrawal [12, 57], Visual Analog Scale [12, 39] and Marijuana Withdrawal Scale [53, 54] were all used in two different studies. Single studies reported using a self‐report of subjective withdrawal symptoms (unspecified scale) [58], psychiatrist reports based on physical examinations [22], self‐report via the Alcohol Withdrawal Symptoms Score [39] and self‐report via Severity of Withdrawal Scale [59].

##### Relapse/return to drug use at follow‐up

Thirty studies reported outcomes related to relapse/drug use at follow‐up using a variety of 20 self‐report and 12 objective measures. Self‐reported measures included interviews such as the Interview for Research on Addictive Behaviour [60] and self‐administered questionnaires such as the Alcohol Use Disorders Identification Test [26, 61], Drug Use Disorders Identification Test and the Drug Use Disorders Identification Test‐Extended E [62], the Cannabis Problems Questionnaire and the Short Form‐12 [56] and the Severity of Dependence Scale [56, 63]. Assessments took place at different intervals, ranging from 2 weeks [14, 24, 56] to 3 years after discharge [64]. Objective measures of relapse included positive urinalysis results and readmission to hospitals or inpatient withdrawal treatment. The time‐frame for readmissions varied greatly from within 30 days of discharge [25] to within 2.5 years of discharge [65]. Other outcomes related to relapse include the use of a Maudsley Addiction Profile and author‐created questionnaire to determine factors related to relapse in one study [66] and measuring the effect of social factors on relapse in another [67].

##### Psychological symptoms

Twenty‐four studies reported outcomes relating to psychological symptoms. Twenty‐three different self‐report measures were used. The most common was the Beck Depression Inventory first or second edition (10 studies, [26, 27, 34, 47, 48, 49, 51, 61, 68, 69]). Depression was one of the most assessed psychological constructs and was also measured in other studies using aspects of other measures, such as the Depression, Anxiety and Stress Scale—21 Items [56, 59, 70], Hamilton Depression Rating Scale [39, 71] and Hospital Anxiety and Depression Scale [44]. The next most frequently used measures were the Addiction Severity Index or EuropASI (European version of Addiction Severity Index) [28, 72, 73] and State–Trait Anxiety Inventory‐Form Y or State–Trait Anxiety Inventory [26, 49, 61].

##### Retention and dropout

Thirty studies reported outcomes related to treatment retention and dropout. However, they differ widely in their definitions of both. Dropping out of treatment was defined simply as discharge against medical advice in five studies [4, 6, 25, 44, 75]. One study defined treatment dropout as staying less than 4 days in a 2‐week inpatient program [29], another study as a premature self‐discharge, withdrawal of consent for study participation, or development of a relevant comorbidity needing intervention and stabilisation [54] and another study as not completing the entire study trial [52]. Yet another study defined treatment dropout as discharge against medical advice or exclusion due to complications or administrative failure [39]. Dropout was additionally defined simply as non‐completion in two studies [16, 27] and discontinuation of intervention due to assessment refusal or dropout in another study [59]. Similarly, treatment retention was regarded as non‐premature discharge in 13 studies [9, 14, 24, 34, 35, 41, 53, 56, 71, 76, 77, 78] or for 1 study, non‐premature discharge in addition to number of days patients remained in the study [33]. Additionally, two studies defined retention as completing the entire withdrawal treatment process (5 days [12]; 6 days [36]) while another defined retention as remaining enrolled at the end of the 7‐day taper [32]. Other outcomes related to treatment retention include factors (e.g., increased age, personal obligations, etc.) associated with leaving against medical advice in one study [79], predictors of treatment dropout in one study [9] and factors relating to successful withdrawal treatment in another [76].

##### Length of stay

Similar to retention measures, 15 studies reported outcomes related to length of stay. The main difference between retention and length of stay was that there were no predetermined expectations about the required length of treatment (e.g., at least 7 days). Seven studies included outcomes relating to total length of stay in the facility [4, 6, 22, 24, 29, 40, 80]. Eight studies included outcomes relating to length of withdrawal treatment only [40, 41, 53, 54, 71, 72, 76].

##### Biological measures

Eighteen studies reported outcomes related to biological measures. In four studies, serum Brain Derived Neurotrophic Factor levels were measured [43, 81, 82, 84]. In two studies each, researchers measured serum tetrahydrocannabinol levels [53, 56], serum 1‐nor‐9‐carboxy‐delta‐9‐tetrahydrocannabinol levels [54, 56] and DNA methylation levels [84, 85]. In one study each, researchers assessed pupil diameter via a pupilometer [31], heart rate variability measured via sleep electroencephalogram [86], platelet counts (predictive of delirium tremens in alcohol withdrawal) [87], plasma total homocysteine concentrations (predictive of alcohol withdrawal seizures, short‐term cognitive deficits during withdrawal and long‐term cerebral atrophy) and blood concentrations of thiamine, riboflavin and pyridoxine [88], plasma oxytocin levels [56], Th1 and Th17‐related cytokine levels in blood [48], PEth serum levels (indicating chronic heavy drinking), aspartate transaminase, alanine transaminase, gamma‐glutamyl transferase levels (liver function indicators) and complete blood count [45], plasma neurotrophic factor levels [65], blood protein content, protein thiol content, protein carbonylation, reduced glutathione and total reactive antioxidant potential [51], and blood and urine biomarkers' associated with withdrawal/relapse/clinical scales (i.e., CDT and EtG, cytokines and growth factors, antioxidant enzymes, oxidative stress markers and neurochemical markers) [10].

##### Craving

Seventeen studies reported outcomes related to craving. There was a range of different methods used to assess craving including both self‐reported and objective measures. Measures used to assess craving most frequently used a visual analogue scale, utilised in eight studies [10, 12, 33, 34, 39, 46, 59, 77]. The next most frequent measure was the Penn Alcohol Craving Scale (four studies [45, 46, 89, 90]). Another nine craving orientated measures were used in one study each.

##### Withdrawal complications

Ten studies reported outcomes related to safety in terms of withdrawal complications or adverse events during withdrawal treatment, though the operationalisation of this differed widely between studies. Definitions of complications or adverse events include unspecified adverse events in two studies [29, 33]. In one study each, definitions include seizure, death or delirium [4], needing clinical intervention, referral to a medical or emergency team or death, during or within 72 h of stopping withdrawal treatment [6], events according to the lithium adverse event checklist [56] seizure, falls, delirium or requiring sedation that resulted in withheld doses of drug taper [25], side effects reported during physical examination [47], adverse events assessed via the Systematic Assessment for Treatment Emergent Events [77], delirium tremens, seizures or hallucinations [40], and epileptic seizures, hallucinations, and delirium tremens or pre‐delirium [41].

##### Follow‐up treatment

Eleven studies reported outcomes related to follow‐up treatment. Five studies operationalised follow‐up treatment as attendance of at least one treatment session after discharge from withdrawal treatment, but the time periods in which treatment had to be attended varied from within 14 days of discharge [30] to within 6 months after intake [91]. Yet another study operationalised it as attending one or five follow‐up treatment sessions within a 1‐year follow‐up period [29]. In one study each, follow‐up treatment has been operationalised as remaining in follow‐up treatment at 6‐month follow up [11], time to entry into follow‐up treatment and number of days in follow‐up treatment at 1, 3 and 6 months [91], prescribed buprenorphine and methadone use in the 30 days prior to 1‐, 3‐ and 6‐month follow‐up [92], contacting an outpatient alcohol treatment clinic within 30 days of discharge and remaining in outpatient treatment at 3 months after discharge [93], use of the clinic's patient transport/taxi to the follow‐up residential or day‐clinic for alcohol use disorders and/or co‐morbid disorder treatment (indicative of utilisation of follow‐up care) [27], induction onto extended‐release naltrexone after withdrawal treatment [94], 12‐step group affiliation (i.e., meeting attendance and 12‐step group involvement) assessed via the Alcoholics Anonymous Affiliation Scale [95] and self‐reported attendance at 12‐step meetings or outpatient treatment [28].

##### Sleep

Six studies reported outcomes related to sleep during withdrawal treatment. Three studies used Actigraphy, an objective measure [37, 44, 55], two studies used self‐report measures, such as the Pittsburgh Sleep Quality Index [89, 96], and two used the Sleep‐Related Behaviours Questionnaire [44, 89]. Another eight separate studies used eight different measures, including beliefs and attitudes about sleep, hours of sleep and other sleep details (e.g., onset latency, wake time, etc.).

##### Cognitive measures

Five studies reported outcomes related to cognitive measures. The most frequently used measure was the digit span test in two studies [34, 69] and the Stroop test in two studies [69, 97]. Another two measures were used in only single studies, and these included behavioural and electrophysiological responses to cue reactivity (visual oddball paradigm) and inhibition tasks (go/no‐go task) [98].

##### General health

Four studies reported outcomes concerning overall health (e.g., aspects of physical and psychological health, quality of life). The measures were self‐report and assessed aspects of both physical and psychological health. The World Health Organization Quality of Life scale was used in two studies [56, 61], the Short Form‐12 was used in one study [56] and the RAND Medical Outcomes Study Short Form Health Survey (Short Form‐36) was used in one study [72].

##### Physical health

Two studies reported outcomes concerning aspects of physical health. These measures were self‐report, including the Charlston‐Comorbidity Index in one study [39] and HIV risk behaviours assessed via the Revised Risk Behaviour Assessment in one study [99].

##### Satisfaction

Four studies reported outcomes related to client satisfaction, all using different self‐report measures. Measures were the Treatment Process Questionnaire (which assessed clients' understanding, satisfaction and perceived benefit of the treatment) [72], acceptability of intervention based on whether it: (i) improved their attention; (ii) reduced their craving for methamphetamine; and (iii) was interesting [14], Client Satisfaction Questionnaire [63] and a general client satisfaction measure [42].

##### Abstinence self‐efficacy

Two studies reported outcomes related to self‐efficacy. Self‐report measures used were the 12‐item versions of the Alcohol Abstinence Self‐Efficacy Scale and Drug Abstinence Self‐Efficacy Scale in one study [62] and the Brief Situational Confidence Questionnaire in another [28].

##### Others

Three studies reported outcomes that did not fit into the above categories. These measures were employment and death observed 11 years after withdrawal treatment in one study [16], motivation to continue treatment assessed via the German short form of the University of Rhode Island Change Assessment and the ‘Veränderungsstadien–Skala’, also known as the Stages of Change Scale, in one study [27], and self‐reported religious coping via the Brief Measure of Religious Coping in one study [100].

## 4 DISCUSSION

Overall, the assessment of withdrawal treatment outcomes seems to mainly concern psychological (e.g., craving, mental health) and biological markers (e.g., withdrawal symptom severity, biomarkers), with social outcomes (e.g., homelessness, quality of social or family relationships) very rarely measured. The results indicate that the field of withdrawal treatment research is concerned with the assessment of withdrawal treatment outcomes beyond abstinence alone. Thus, our results align with research showing that withdrawal treatment services consider outcomes beyond abstinence as important [101, 102]. One conceptual framework of recovery proposes seven aspects that should be measured: physical, biological (i.e., biomarkers), psychological, psychiatric, family, social and spiritual aspects as well as chemical dependence [103]. The SAMHSA recovery model [21] also promotes a holistic view of recovery that includes biological, psychological and social functioning. Another framework for measuring substance use outcomes holistically is the Australian Treatment Outcomes Profile which measures risk, health and wellbeing, and current substance use for those receiving treatment [104]. If such frameworks extend to withdrawal treatment services, then results from the current review clearly indicate that additional measures of social outcomes are warranted. Further support for the measurement of social outcomes comes from a survey of alcohol and drug users and their loved ones, which revealed that, in addition to abstinence and health, improved social circumstances (e.g., stable housing), supportive relationships, confidence, and wellbeing of family and friends were also seen as desirable outcomes for withdrawal treatment [102].

Despite few measures of social outcomes, the review revealed an extremely wide range of outcomes that researchers use to determine the outcomes of withdrawal treatment. Even within particular domains, diverse measures were used. For example, craving was measured in 17 different studies using 21 different instruments, spanning both self‐reported and objective measures, as well as different questionnaires. There is a stark lack of consensus regarding measurements, even within a single domain, which makes it difficult for clinicians and professionals to compare findings or evaluate relative effectiveness of withdrawal treatment services. This is particularly important when trying to understand the relative merits of withdrawal treatment services of varying lengths, content and for different drug types.

The review reveals that in some areas (e.g., psychological symptoms distress) there are multiple measures that have satisfactory psychometric characteristics to choose from. This may require some consensus at least within specific domains such as the assessment of depressive symptoms. Similarly, greater clarity regarding criteria for determining dropout and/or reporting retention is needed. It is unlikely that consensus could be found for measures in all domains, but it may be possible to obtain some agreement for particular domains. Examples of such efforts include the Outcome Reporting in Brief Intervention Trials: Alcohol (ORBITAL) Core Outcome Set [105, 106]. The ORBITAL Core Outcome Set proposes a set of outcome domains to measure in interventions as well as which tools should be used for assessment. For example, it proposes that recent alcohol consumption should be measured as the total number of standard drinks consumed within the last week, in grams, via timeline follow back and using a standard drink guide. The ORBITAL recommendations are composed entirely of self‐report measures and although it has been acknowledged that self‐reports carry risk of under‐reporting [107], the authors of the ORBITAL recommendations argue that objective measures such as biomarkers are not accessible in every country. Thus, the inclusion of objective measures would not be viable in creating a list of outcomes that is accessible to all studies [106].

Not all of the ORBITAL recommendations are suitable for measuring outcomes immediately after withdrawal treatment since most programs require strict abstinence. Further, ORBITAL was designed for alcohol use [106]. Thus, development of recommended outcomes and associated measures for withdrawal treatment would necessitate additional measures and methods that focus specifically on outcomes from withdrawal treatment services. The current review provides a starting resource for such a process.

Another finding from the literature review was a paucity of studies assessing client satisfaction as a withdrawal treatment outcome. Indeed, only 4 of the 88 studies in this review attempted to measure client satisfaction. Similar to other outcome domains, each of these four studies measured client satisfaction differently. The lack of assessment of client satisfaction and experience across studies looking at withdrawal treatment outcomes is concerning given the positive correlation between client satisfaction and treatment completion [108] as well as longer‐term treatment outcomes [109].

Similarly, other key social outcomes (e.g., housing) highlighted in more recent recovery frameworks [21, 103] were rarely assessed. Access to stable accommodation has been highlighted as one important component of recovery [102]. Homelessness has been linked to non‐attendance at follow‐up treatment after discharge from withdrawal treatment [8] and increased re‐admission rates to withdrawal treatment [110]. Thus, assessing whether accommodation needs have been satisfactorily addressed during withdrawal treatment is an area of outcome assessment worthy of increased attention. Finally, future research should investigate levels of treatment success in inpatient withdrawal treatment settings.

### 4.1 Limitations

The most significant limitation of this review was its scope. The analysis was restricted to articles published in English in 2012 or later and six databases. Additionally, our decision to restrict the scope of this article to only inpatient withdrawal treatment programs could limit the generalisability of our results to other types of withdrawal treatment programs, such as outpatient withdrawal treatment programs. However, the limited scope to studies published in 2012 and later allowed the review to focus on outcomes subsequent to more recent developments in the field, particularly recovery frameworks (e.g., [21]).

This review includes studies from multiple countries around the world with differing healthcare systems (e.g., universal health‐care systems, insurance systems, etc.), which could impact both the types of withdrawal treatment outcomes assessed as well as the results of these outcomes.

## 5 CONCLUSION

This literature review has highlighted three important aspects of withdrawal treatment outcome measurement that should be addressed. First, there needs to be greater consensus about the outcomes domains that should be assessed. Second, within domains, a narrower range of measures could be recommended. This might involve a review and consensus process similar to ORBITAL [103, 106]. Third, there are several social outcome domains that are rarely assessed but given their importance in recent consumer and recovery definitions, are clearly worthy of inclusion in future research (e.g., client satisfaction, social/family relationships, housing).

## AUTHOR CONTRIBUTIONS

Each author certifies that their contribution to this work meets the standards of the International Committee of Medical Journal Editors.

## CONFLICT OF INTEREST

No external funding was received by any authors for this study.

## Supporting information


**Table 1.** Summary of withdrawal treatment outcomes in inpatient settings for alcohol users
**Table 2.** Summary of withdrawal treatment outcomes in inpatient settings for opioid users
**Table 3.** Summary of withdrawal treatment outcomes in inpatient settings for other drug users
