# Supplementary material for: A narrative review of outcome measures used in drug and alcohol inpatient withdrawal treatment research
Source: Drug Alcohol Rev. 2023 Jan 12;42(2):415–26. doi: 10.1111/dar.13591 (PMC10108086; doi:10.1111/dar.13591)
Supplement: Supplementary file 1 — Table 1. Summary of withdrawal treatment outcomes in inpatient settings for alcohol users Table 2. Summary of withdrawal treatment outcomes in inpatient settings for opioid users Table 3. Summary of withdrawal treatment outcomes in inpatient settings for other drug users [file DAR-42-415-s001.docx]

Table 1. Summary of withdrawal treatment outcomes in inpatient settings for alcohol users

|  | **Author (year), country [ref]** | **Sample** | **Program components** | **Outcome measures** | **Main findings** | **% Complete** | **% Transfer** |  |
| --- | --- | --- | --- | --- | --- | --- | --- | --- |
| 1 | Azuar et al. (2016), France [29] | 120 inpatients with alcohol dependence (80% male, mean age 48.5 years) | Two-week inpatient program.  Treatment of medical conditions associated with alcohol dependence and psychoeducation to prevent relapse.  Quasi-experimental design (two groups): Hospitalised directly from emergency department or hospitalised after motivational preparation before scheduled admission. | - Length of stay.  - Rate of treatment drop out.  - Attended at least 1 post-discharge follow-up.  - Attended at least 5 post-discharge follow-ups. | - Patients hospitalised directly from the emergency department had longer stays than scheduled admission patients.  - For both groups, rate of drop out, attendance for at least 1 follow-up, and attendance for at least 5 follow-ups were the same.  - Mean number of follow-up visits were the same for both groups. | 98.3 | 57-65 |  |
| 2 | Beyraghi et al. (2020), Iran [4] | 83 adult patients with AUD (95.2% male, mean age 45.35) | Symptom-triggered protocol with benzodiazepines as main pharmacological intervention; lorazepam used for patients with severe liver problems or comorbidities | - Completion of inpatient treatment.  - Length of stay.  - Complications. | - 81.7% completed inpatient treatment.  - Mean length of stay was 9.01 days.  - Withdrawal delirium occurred in 7.2% of patients; no withdrawal seizures or death occurred. | 81.7 | NR |  |
| 3 | Brooks et al. (2016), USA [89] | 33 adults over 18 years old seeking treatment for alcohol dependence.  Mean age not reported. | Inpatient treatment lasted 6 weeks or more, followed by 6 weeks of optional treatment as outpatient.  Physical evaluations, inpatient withdrawal management, psychosocial management, education. | - Measured 1 week before discharge and 4-6 weeks after discharge:  - Sleep quality (PSQI) and daytime sleepiness (ESS);  - Sleep related beliefs and behaviours (DBAS-16; Self-Efficacy For Sleep Scale; SRBQ);  - Interviews examining patients’ support system; barriers and facilitators to sleep and abstinence;  - Alcohol craving and relapse 4-6 weeks after discharge (PACS; Timeline Follow-Back). | - 72.7% reported fear or anxiety regarding discharge.  - 82.1% reported difficulties in assimilating back to daily life at post-discharge interview.  - Co-morbidity between alcohol use and sleep disturbances  - Those who reported no sleep disturbances also reported the lowest craving scores. | NR | NR |  |
| 4 | Campanella et al. (2020), Belgium [98] | 65 inpatients with alcohol dependence (72.3% male, aged 25 to 71 years).  25 patients’ data contaminated and discarded; final sample size of 40. | 4-week program.  Fixed schedule of diazepam, group therapy for relapse prevention, psychosocial support, and discharge preparation, and sophrology sessions. | - Abstinence/relapse over a 3-month follow-up.  - Behavioural and electrophysiological responses to cue reactivity and inhibition tasks | - 62.5% relapsed.  - Go P3d amplitude indicates abstinence via devaluation of alcohol-related cues; no-go P3d amplitude indicates relapse via altered inhibitory process.  - Preserved oddball P3d response to alcohol-related cues and increased no-go P3d response in no-go trials in alcohol-related settings related to abstinence.  - In the cue reactivity task, abstainers were more able to accurately detect alcohol cues (alcohol stimuli highly salient).  - In the inhibition task, abstainers showed increased no-go P3d response at session 2 than session 1 in alcohol-related settings (i.e., abstainers more able to inhibit responses than relapsers). | NR | NR |  |
| 5 | Coccini et al. (2021), Italy [10] | 55 adults with alcohol dependence (69% male, median age 50 years) | 28-day program.  Medical management of WS using diazepam, psychological interventions, physical rehabilitation exercises. | - Craving/temptation to drink.  - Anxiety, depression, well-being, perception of positive change via Cognitive Behavioural Assessment-Outcome Evaluation.  - Relapse after 1 month.  - Relationship between biomarkers and clinical scales, relapse, withdrawal. | - Temptation to drink, anxiety and depression decreased from intake to discharge.  - Well-being and perception of positive change increased from intake to discharge.  - MCP1, F2-IsoPs and SOD1 were indicated AUD, withdrawal, relapse.  - SOD1 and well-being correlated with reduced risk of relapse one month post-discharge. | NR | NR |  |
| 6 | Förg et al. (2012), Germany [39] | 42 inpatients with alcohol dependence (71.4% male, mean age 43.9 years) | 7-day program.  Randomised to either pregabalin or placebo.  Diazepam used as rescue medication according to a symptom-triggered protocol. | - Total amount of diazepam administered as rescue medication in each group.  - Difference in AWSS and CIWA-Ar scores between intake and discharge.  - Adverse events.  - Dropout rates.  - Changes in neuropsychological scales from intake to discharge (CCI, HAMA, HAMD, ADS, VAS Withdrawal, VAS Craving). | - 2 patients signed out against medical advice (1 per group); another 4 patients excluded due to complications or administrative failure.  - Diazepam use decreased from intake to discharge (both groups).  - No differences between amount of diazepam administered between groups.  - AWSS and CIWA-Ar scores decreased from intake to discharge.  - Improvements in anxiety, depression and craving from intake to discharge (both groups); no between group differences.  - 16 patients per group reported adverse events during detox, none severe. | 95.2 | NR |  |
| 7 | Ganesha et al. (2013), India [86] | 20 male patients with alcohol dependence (mean age 38.8 years) and 18 controls (matched for age and gender; mean age 39.7 years) | Detox using diazepam.  Detox completed within 48 hours, followed by wash out period (5 half-lives). | - Heart rate variability (obtained via sleep electrocardiogram) on day 8 | - Patients with alcohol dependence had lower heart rate variability than controls.  - Those with highest exposure to alcohol had the lowest scores on both time and frequency measures of heart rate variability. | NR | NR |  |
| 8 | Harshe et al. (2017), India [87] | 40 patients with alcohol dependence (100% male, mean age 38.5 years) | Detox with lorazepam and thiamine; lorazepam tapered gradually from day 6 and stopped completely by discharge | - Platelet counts on 5 days of blood collection (day 2, 4, 6, 8, 10) | - Patients with delirium tremens had lower platelet counts than those without delirium tremens at all measurements.  - Mean platelet counts gradually rose on days 6, 8 and 10.  - Patients with delirium tremens had a higher increase in platelet counts on day 8 and 10 compared to those without delirium tremens. | NR | NR |  |
| 9 | Heese et al. (2012), Germany [88] | 168 patients with alcohol dependence (65.5% male, mean age 48.1 years) | Detox with clomethiazole according to symptom-triggered protocol; benzodiazepines used if clomethiazole could not be used. | - Plasma total HCY concentrations on day 1, 7, 11.  - Blood concentrations of thiamine, riboflavin and pyridoxine on day 1, 7, 11. | - Elevated HCY levels in patients undergoing alcohol detox  - Blood alcohol levels on admission, folate serum levels and riboflavin serum levels affected HCY levels over time | NR | NR |  |
| 10 | Hufnagel et al. (2017), Germany [111] | 300 patients with alcohol dependence (66.2% male, mean age 46.3 years) | Detox as usual vs. motivational interviewing plus psychoeducation in addition to regular detox.  During detox, patients received clomethiazol or diazepam as needed and levetiracetam, if they had a history of withdrawal seizures. | - Alcohol and tobacco use at follow-up (3, 6, 12 months after discharge) | - Baseline smoking status at detox intake is associated with drinking outcomes.  - Smokers had higher risk of relapse in alcohol consumption during the first year after detox. | NR | NR |  |
| 11 | Janakiraman et al. (2020), USA [90] | 146 patients with AUD (70.5% male, median age 50.4 years) | Benzodiazepines during the first week of inpatient treatment if necessary | - PACS on day 5, 12, 19, 26 (analysis only included first 3 days due to attrition) | - Craving decreased across time.  - Those with past suicide attempt had higher craving levels than those without suicidal history (ideation or attempt). | NR | NR |  |
| 12 | Kethawath et al. (2020), India [43] | 25 male inpatients with alcohol dependence (mean age 38.2 years) and 25 male controls (mean age 36.0 years) | 7-10 days, benzodiazepine taper | - Serum BDNF levels from blood samples drawn on day 1, 5 and 10.  - CIWA-Ar on day 1, 5 and 10 to assess WS. | - Patients showed significant reduction in CIWA-Ar scores from day 1 to day 10.  - BDNF levels increased from baseline to day 10.  - No difference in BDNF levels between patients and controls. | NR | NR |  |
| 13 | Koller et al. (2019), Germany [84] | 23 alcohol-dependent inpatients (100% male, mean age 49.04 years) and 13 healthy controls (100% male, mean age 41.3 years) | 15-day program: somatic detox, individual and group psychotherapy, counselling regarding social and financial problems, somatic medical care.  All patients received pharmacological treatment with oxazepam based on withdrawal severity. | - Blood samples taken at intake, after 7 days and after 15 days to analyse DNA methylation | - Increase in global methylation during withdrawal in alcohol-dependent patients; difference between them and controls increased over time.  - Hydroxymethylation significantly lower in alcohol-dependent patients than controls at all time points, but this difference decreased over time. | NR | NR |  |
| 14 | Lucht et al. (2021), Germany [112] | 462 patients with alcohol dependence.  TAU + SMS group: 77.4% male, mean age 45.4 years.  TAU group: 77.2% male, mean age 44.5 years | Randomised to TAU or TAU + SMS intervention.  2-week program, including psychosocial treatment. | - Alcohol consumption 10-12 months after discharge | - Attrition in follow-up period higher in TAU than TAU+SMS group.  - In the TAU+SMS group, 22.2% were heavy drinkers at follow-up compared to 32.3% in TAU group.  - TAU+SMS group had more abstinence days than TAU group. | NR |  |  |
| 15 | Neu et al. (2018), Germany [96] | 77 alcohol-dependent inpatients (76.6% male, mean age 47.7 years) | Medical management of WS using clomethiazole in addition to psychoeducation and relapse prevention | - Self-reported sleep quality assessed via PSQI | - Sleep quality of participants improved significantly, no gender effects, no effect of age.  - Patients who had bad sleep at intake improved more than patients who had better sleep at intake.  - No difference in PSQI score of those who did not complete treatment and those who completed treatment | NR | NR |  |
| 16 | Nichols et al. (2019), USA [40] | 83 alcohol-dependent inpatients: Gabapentin group (*n* = 40): 63% male, mean age 42 years; Control group (*n* = 43): 70% male, mean age 46 years | Gabapentin group: Gabapentin and symptom-triggered benzodiazepines (mostly lorazepam, rarely diazepam).  Control: Symptom-triggered benzodiazepines only. | - Impact of gabapentin use on the use of as-needed benzodiazepines.  - Duration of alcohol withdrawal protocol (no. of days with active CIWA-Ar protocol).  - Length of stay.  - Withdrawal complications. | - Gabapentin did not affect duration of alcohol withdrawal protocol, use of as-needed benzodiazepines, length of stay.  - Gabapentin group had more complicated withdrawal instances but difference not statistically significant. | NR | NR |  |
| 17 | Nielsen & Nielsen (2018), Denmar [93]k | 100 alcohol-dependent inpatients:  TAU (*n* = 55): 76.4% male, mean age 49.7 years; Outreach (*n* = 45): 57.8% male, mean age 45.7 years | Maximum 10 days’ detox with benzodiazepines.  Randomly allocated to: TAU: Referred to treatment at affiliated outpatient alcohol treatment clinic; Outreach: Motivational interview with therapist and agreement on attendance at affiliated outpatient alcohol treatment clinic. | - Percentage of patients who turned up the affiliated alcohol treatment clinic within 30 days of discharge.  - Number of patients remaining in outpatient treatment at 3 months post-discharge.  - Admission to mental health and somatic hospitals 12 months post-discharge | - More patients from outreach group turned up to affiliated alcohol treatment clinic than TAU group (84.4% vs. 58.2%).  - More patients in outreach group remained in treatment at 3 months compared to TAU group (46.7% vs. 23.6%).  - No group differences in admission to hospitals 12 months after discharge | NR | 58.2-84.4 |  |
| 18 | Olivia et al. (2018), Italy [59] | 187 alcohol-dependent patients (68.5% male, mean age 46.6 years) | 1-week fixed schedule protocol using medication with various therapies (e.g., individual and group psychotherapy, motivational enhancement therapy, nutrition education) included at clinician’s discretion | - Assessments at baseline (T0), discharge (T1), 6 months (T2) and 12 months (T3) after discharge.  - All follow-up assessments measured alcohol use severity (AUDIT), state and trait anxiety (STAI-Y) and depression (BDI) | - At T2, 47.6% of 164 patients available for follow-up had relapsed (no gender differences); of these patients, 32.9% had high risk, 3.7% had hazardous drinking, 7.3% were excessive drinkers and 3.7% had low health risk, according to AUDIT scores.  - At T3, 53.4% of 146 patients available for follow-up had relapsed, of which 9.6% had relapsed between T2 and T3; no differences in terms of AUDIT domains.  - At T0 & T3, females showed higher state and trait anxiety and depression but same alcohol use severity compared to males.  - Both genders improved in terms of state and trait anxiety and depression (T1, T2, T3), but females showed more improvement. | NR | NR |  |
| 19 | Ooms et al. (2021), Netherlands [27] | 52 alcohol-dependent inpatients (31.2% male, mean age 45.0 years) | Detox completed within 3 weeks, usually with chlordiazepoxide | - Self-reported severity of WS using Dutch “Severity of Withdrawal Scale”.  - Alcohol craving assessed using VAS.  - DAQ.  - DASS-21. | - Different withdrawal patterns during detox for patients with more vs. less than 2 previous detox—the former has higher subjective withdrawal symptoms at intake but not discharge; in general, more than 2 previous detox predicts more severe withdrawal.  - Those with more than 2 previous detox had worse scores on all DASS-21 domains than those with less than 2 detox.  - No group differences for DAQ and VAS between admission and discharge.  - No group differences at 1-month follow up. | 84.6 | NR |  |
| 20 | Ostergaard et al. (2018), Germany [68] | 122 alcohol-dependent patients (71.3% male, mean age 45.79 years).  77 in the intervention condition (33 in PAST and 44 in PASD), 45 in control. | TAU (psychiatric care and psychopharmacological care) plus either intervention (PAST or PASD) or control.  PAST (for AUD and PTSD) and PASD (for AUD and major depression): Recognise co-occurrence of AUD with other diagnosis, to exchange coping experiences, motivate detox completion and enrol in follow-up treatment. | - Assessments were conducted about 8.55 days after admission (T1) and 10 days after the first assessment (T2).  - Treatment motivation via German short form of URICA and VSS-K.  - Readmission in the 6 months after discharge.  - Utilisation of follow-up care (use of the clinic’s patient transport/taxi to the follow up residential or day-clinic for AUD and/or co-morbid disorder treatment). | - Of the 87 patients who had data available for URICA at T1 and T2, there was no effect of intervention on URICA.  - 77.87% of the 122 patients in the study had no readmissions in the follow-up period; of those that were re-admitted, more were from the control group than the intervention groups.  - 43.44% were directly transferred into follow-up care; those in intervention condition were more likely to enrol into follow-up care than controls.  - Intervention predicted utilisation of follow-up care.  - More severe depression predicted utilisation of follow-up care in the intervention groups but not the controls.  - Being in intervention predicted utilisation of follow-up care if patients had high trauma scores. | 71.3 | 43.4 |  |
| 21 | Ostergaard et al. (2019), Germany [46] | 87 alcohol-dependent patients (74.7% male, mean age 45.22 years) | Same as above Ostergaard et al. (2018) | - Self-reported depression (BDI-II).  - Self-reported PTSD symptoms (German version of the PTSD Checklist for DSM-5; PCL-5). | - BDI-II scores decreased from baseline to discharge but there was no effect of intervention.  - All PTSD domains decreased from baseline to discharge, avoidance and hyperarousal domains showed more reduction than re-experiencing and negative cognition domains. | NR | NR |  |
| 22 | Pedersen et al. (2013), USA [69] | 11 alcohol-dependent patients (81.8% male, mean age 42.27 years) | Intranasal oxytocin or placebo given two times a day for 3 days in patients undergoing symptom-driven PRN lorazepam detox | - CIWA scores.  - AWSC.  - On day 2 and 3 also completed PACS, ACVAS and POMS first edition. | - Compared to placebo, oxytocin group needed less total lorazepam to complete detox, had lower CIWA scores than placebo.  - Oxytocin group lower AWSC scores on day 1, almost significant on day 2.  - Alcohol craving scores were almost lower (PACS) or were significantly lower (ACVAS) in oxytocin group than placebo on day 2 but not day 3.  - POMS Tension/Anxiety domain scores significantly lower in oxytocin group on day 2 but not day 3 | NR | NR |  |
| 23 | Petit et al. (2017), Belgium [61] | 41 alcohol dependent inpatients (73.2% male, mean age 49.54 years) and 41 controls (58.5% male, mean age 43.80 years) | 18 days’ detox via benzodiazepines, tapered gradually | - Craving self-reported via OCDS.  - Self-reported mood states via PANAS and BDI.  - Cognitive flexibility (Number-Letter task).  - Verbal fluency (Word fluency tests).  - Inhibition (Stroop task).  - Short-term memory (Digit span task).  - Working memory (modified Brown-Peterson task). | - Craving.  - Alcohol-dependent patients performed worse than controls in the letter portion of the cognitive flexibility task.  - No difference in groups regarding verbal fluency.  - Alcohol-dependent patients had impaired inhibition and working memory at baseline but not at the end of detox.  - Decrease in craving and depressive scores and increase in positive affect from baseline to end of detox, but no correlation with improvements in working memory task. | NR | NR |  |
| 24 | Picci et al. (2014), Italy [80] | Alcohol dependence.  199 enrolled but only 146 responders.  Of these 146 responders, 68.5% were male, mean age 46.6 years. | 7-day program.  Detox with lorazepam or oxazepam or another benzodiazepine and tapering dosage from day 4 to day 7.  Non-pharmacological treatments (parent training intervention, short-term individual psychotherapy, group psychotherapy, motivational enhancement therapy, nutrition education) included at clinician’s discretion. | - Measurements taken at day 7 (T0), 6 months after discharge (T1), 12 months after discharge (T2).  - AUDIT for severity of alcohol use.  - WHOQOL-BREF for quality of life.  - STAI-Y and BDI for anxiety and depressive symptoms respectively. | - At T1, 47% of 168 contactable patients had relapsed; AUDIT scores indicate 32.7% at high health risk (risk zone 4), 3.6% in risk zone 3, 7.1% in risk zone 2, 3.6% at low health risk (risk zone 1).  - At T2, 43.8% of 168 patients had relapsed; AUDIT scores indicate 34.2% at high health risk (risk zone 4), 3.4% in risk zone 3, 4.1% in risk zone 2, 2% at low health risk (risk zone 1).  - 9.6% who relapsed at T1 were abstinent 6 months later; 7.53% of those who were abstinent at T1 later relapsed at T2.  - At T0, no difference in WHOQOL scores between abstinent and relapsed group.  - At T1, abstinent group reported improvements in WHOQOL scores but relapsed group reported no improvements.  - T2 changes in WHOQOL scores had no group differences; increase in values with regards to physical and psychological health and social relationships. | NR | NR |  |
| 25 | Quelch et al. (2019), United Kingdom [44] | 40 alcohol dependent inpatients from two different detox sites.  WMPU: 20 patients, 75% male, mean age 47.5 years.  UHB: 20 patients, 75% male, mean age 50.2 years | - WMPU site: Symptom-triggered approach based on CIWA-Ar scores; referred to outpatient follow-up care; offered outpatient follow up after discharge for ongoing support.  - UHB site: Fixed dose benzodiazepine regimen and referral to community outreach services but no outpatient follow-up. | - No. of emergency hospital readmissions with alcohol withdrawal or intoxication (taken to indicate relapse).  - Length of stay.  - Time to first re-admission.  - No. of re-admissions. | - No difference in length of stay between two sites.  - 100% of UHB patients were readmitted after detox vs. 40% of WMPU patients.  - Length of time to first readmission longer for WMPU but not significant due to small sample.  - Readmissions per patient and total number of readmissions at UHB is more than WMPU. | NR | NR |  |
| 26 | Smith et al. (2014), United Kingdom [45] | 71 alcohol-dependent inpatients (64.8% male, mean age 42.8 years) | Anxiolytic medication in the first 7-9 days of detox | - Sleep diary completed over a 7-day period: sleep onset latency, wake time after sleep onset, total sleep time.  - Actigraph for activity readings and sleep data.  - GCTI.  - DBAS-10.  - SRBQ.  - Relapse in the past month since discharge.  - HADS.  - CIWA-Ar. | - 8.5% reported scores above the cut-off on the CIWA-Ar, indicating WS that require medical intervention.  - CIWA-Ar scores correlated with HADS anxiety and depression domains.  - HADS measures correlated with DBAS, GCTI and SRBQ.  - CIWA-Ar correlated with GCTI and SRBQ.  - Of 62 who could be contacted at follow-up, 69.4% had relapsed.  - DBAS-10 scores were higher in non-relapsers.  - Sleep onset latency longer in those who relapsed. | NR | NR | |
| 27 | Sonmez et al. (2017), Turkey [41] | 54 male participants: 22 alcohol-dependent patients (mean age 45.7 years); 32 healthy controls (16 non-drinkers, mean age 43.9 years; 16 social drinkers, mean age 39.7 years) | 14-day diazepam detox, dose based on withdrawal symptom severity.  No other psychopharmacological agents used during detox. | - Blood samples taken on day 1, 7, 14 to measure PEth serum levels (indicating chronic heavy drinking), as well as AST, ALT, GCT (liver function indicators) and complete blood count.  - PACS to measure alcohol craving on day 1, 7, 14.  - CIWA-Ar to measure alcohol withdrawal on day 1, 7, 14. | - PEth levels of patients reduced during alcohol withdrawal; but no difference between days 7 and 14.  - PEth levels of patients did not differ from non-drinkers and social drinkers.  - PEth levels correlated with self-reported drinking amount in the past month.  - PEth levels trended towards significance in terms of correlation with AUDIT scores at baseline.  - PEth levels not correlated with CIWA-Ar, PACS, or total amount of benzodiazepine dose during detox.  - PEth levels not correlated with GCT, MCV, ALT, AST, CIWA-Ar, PACS scores on days 7 and 14. | NR | NR | |
| 28 | Soravia et al. (2018), Switzerland [67] | 301 patients with AUD: TAU group (*n* = 125): 60.8% male, mean age 47.20 years; AWS group (*n* = 176): 72.2% male, mean age 46.96 years | TAU group: Fixed scheme of benzodiazepines.  AWS group: Benzodiazepine dose dependent on AWS scores. | - Treatment duration, medication, complications, costs of detox, treatment drop out | - % of patients being treated with benzodiazepines decreased between TAU to AWS group; decreased dosage of lorazepam and additional withdrawal medication (CPZI) but no difference for dosage of diazepam.  - Time spent in detox (from admission to last benzodiazepine dose) lower in AWS than TAU.  - No differences between AWS and TAU group in terms of detox complications and treatment drop out. | NR | NR |  |
| 29 | Van den Berg et al. (2015), Netherlands [85] | 132 alcohol dependent patients (60.6% male, mean age 63.4 years) | Detox with benzodiazepines | - Readmission within 1 year of discharge.  - Effect of social factors on readmission.  - Number of readmissions.  - Time to first readmission | - 50.8% were readmitted within a year.  - Median no. of readmissions was 2.  - Median time to first readmission was 88 days.  - Spending majority of leisure time alone predicted fewer readmissions (compared to spending leisure time with others). | NR | NR |  |
| 30 | Witt et al., (2020), Germany [52] | 99 alcohol-dependent inpatients (100% male, mean age 47.6 years).  95 age-matched controls (100% male, mean age 47.4 years). | Medically supervised withdrawal management | - DNA methylation (measured during acute withdrawal and after 2 weeks of recovery) | - 2879 CpG sites differentially methylated between acute withdrawal and recovery, including CpG sites that are involved in withdrawal symptoms.  - Methylation differed between patients and controls (9845 and 6094 CpG sites at withdrawal and recovery compared to controls, respectively) at sites that are involved in withdrawal. | NR | NR |  |

*Note.* ACVAS, Alcohol Craving Visual Analog Scales; ADS, Alcohol Dependence Scale; ALT, alanine transaminase; AST, aspartate transaminase; AUD, alcohol use disorder; AUDIT, Alcohol Use Severity Identification Test; AWS, Alcohol Withdrawal Scale; AWSC, Alcohol Withdrawal Symptoms Checklist; AWSS, Alcohol Withdrawal Symptoms Score; BDI, Beck Depression Inventory First Edition; BDI-II, Beck Depression Inventory Second Edition; BDNF, Brain Derived Neurotrophic Factor; CCI, Charlston-Comorbidity Index; CIWA, Clinical Institute Withdrawal Assessment for Alcohol; CIWA-Ar, Clinical Institute Withdrawal Assessment for Alcohol (Revised Version); CpG, Cytosine-Phosphate-Guanine; DAQ, Desire for Alcohol Questionnaire; DASS-21, Depression, Anxiety and Stress Scale – 21 Items; DBAS-10, Dysfunctional Beliefs about Sleep Scale-10 Item Version; ESS, Epworth Sleepiness Scale; GCTI, Glasgow Content of Thoughts; HADS, Hospital Anxiety and Depression Scale; HAMA, Hamilton Anxiety Rating Scale; HAMD, Hamilton Depression Rating Scale; HCY, homocysteine; NR, not reported; OCDS, Obsessive-Compulsive Drinking Scale; PACS, Penn Alcohol Craving Scale; PANAS, Positive Affectivity Negative Affectivity Schedule; PASD, Psychoeducational Group Intervention on Alcohol Drinking Related To Stress And Depression; PAST, Psychoeducational Group Intervention on Alcohol Drinking Related to Stress and Trauma; POMS, Profile Of Mood States; PSQI, Pittsburgh Sleep Quality Index; PTSD, post-traumatic stress disorder; SMS, short service message; SOD, Superoxide Dismutase; SRBQ, Sleep-Related Behaviour Questionnaire; STAI-Y, State-Trait Anxiety Inventory – Form Y; TAU, treatment as usual; UHB, University Hospitals Birmingham; URICA, University of Rhode Island Change Assessment; VAS, Visual Analog Scale; VSS-K, Stages of Change Scale; WHOQOL-BREF, Abbreviated World Health Organization Quality For Life Scale; WMPU, West Midlands Prison Unit; WS, withdrawal severity.

Table 2. Summary of withdrawal treatment outcomes in inpatient settings for opioid users

|  | **Author (year), country** | **Sample** | **Program components** | **Outcome measures** | **Main findings** | **% Complete** | **% Transfer** |
| --- | --- | --- | --- | --- | --- | --- | --- |
| 1 | Amiri et al. (2014), Iran [23] | 69 opioid-dependent patients (100% male, mean age 28.5 years) | Two groups: clonidine + clonazepam + acetaminophen or clonidine + clonazepam + acetaminophen + amantadine | - WS severity at baseline and after 24h, 48h and 72h, using COWS.  - Completion rate. | - Patients receiving amantadine had milder WS.  - Severity of WS increased across time in both groups.  - 30 per group completed the trial. | 87.0 | NR |
| 2 | Baxley et al. (2019), USA [12] | 90 opioid-dependent patients.  Low AS: 45.8% male, mean age 36.4 years.  High AS: 64.3% male, mean age 38.8 years. | 5-day buprenorphine-assisted detox | - Self-reported withdrawal severity via ARSW.  - VAS assessing opioid craving, withdrawal severity and fear of withdrawal.  - Relapse.  - Engagement in follow-up treatment. | - All patients completed the 5-day detox.  - High AS predicted more severe WS and greater fear of withdrawal.  - 68% attended at least 1 day of follow-up treatment, but 65% of these did not remain in follow-up treatment.  - Almost 60% relapsed within 30 days after discharge.  - AS did not predict adherence to follow-up treatment or relapse. | 100.0 | 68 |
| 3 | Behnam et al. (2012), Iran [66] | 60 men with SUD, specifically opioids (mean age 54.1 years) | 7-day program.  Randomised to placebo or gabapentin. | - Opioid withdrawal measured using SOWS daily from day 1 to 7 (specifically used the domain of body pain to assess pain severity) | - Pain severity decreased over time in both groups, with pain severity being significantly lower for the gabapentin group than placebo | NR | NR |
| 4 | Cushman et al. (2016), USA [11] | 113 patients with OUD (69.0% male, mean age 39.5 years) | Two groups:  Detox group: 5-day buprenorphine/naltrexone taper in a 4:1 ratio; given a list of OUD treatment centres for patients to self-refer;  Linkage group: 5-day buprenorphine/naltrexone taper in a 4:1 ratio; scheduled initial visit to primary care buprenorphine clinic. | - Injection opiate use at follow-up (1, 3, 6 months).  - Attendance at follow-up treatment (1, 3, 6 months). | - Injection opiate use decreased at all 3 follow-ups.  - No difference in injection opiate use between groups.  - At 1 month follow-up, 70.6% in linkage group had attended one follow-up treatment session compared to 9.7% in the detox group.  - At 6-month follow-up, 13.7% in linkage group remained in follow-up treatment compared to 3.2% in detox group. | NR | 9.7-70.6 |
| 5 | Ducray et al. (2012), Ireland [31] | 102 opioid dependent patients (65% male, median age of admission 22 years) | 2 weeks of medically assisted detox | - Drug use factors reported during first relapse episode after discharge from residential detox | - 71% relapsed within 1 week of discharge.  - 72% who used heroin were with another drug user during first relapse and 51% injected heroin during this first lapse.  - 65% of those who were alone during their first relapse injected heroin.  - Low mood and craving were the most critical factors in relapse. | NR | NR |
| 6 | Dunn et al. (2017), USA [32] | 103 participants with opioid dependence (85.4% male, mean age 28.9 years) | 26-28 day residential detox with 3 phases: (i) morphine stabilisation; (ii) naloxone challenge; (iii) randomised into clonidine, tramadol ER or buprenorphine taper.  Taper lasted 7 days. | - COWS.  - SOWS.  - Pupil diameter.  - Adverse events. | - Clonidine group experienced more severe WS than tramadol ER and buprenorphine group during taper phase but not the post-taper phases.  - Clonidine and tramadol ER groups (but not buprenorphine group) experienced significant reductions in WS between taper and post-taper phases.  - Pupil diameter increased between stabilisation and taper and post-taper phases for both clonidine and tramadol ER groups; buprenorphine group only had increase in pupil diameter between stabilisation and post-taper phase.  - Adverse events reported by 50-55.6% of the participants (none were serious); most common events were opioid WS. | NR | NR |
| 7 | Dunn et al. (2020), USA [22] | 103 opioid dependent patients (85.4% male, mean age 41.2 years) | Maximum 28 days.  Randomised to buprenorphine, tramadol-ER, or clonidine taper.  Taper lasted 7 days. | - Self-reported and observer ratings of opioid WS (COWS completed by study staff, SOWS completed by participants) | - Lacrimation and vomiting were most (86.8%) and least (37.2%) endorsed symptoms on SOWS.  - Change in resting pulse rate (97.1%) and gooseflesh (19.6%) were most and least endorsed symptoms on the COWS.  - Symptom onset (SOWS) began at mean of 9.3 hours with “feel like using” and ended at mean of 21.0 hours with “cold flashes”.  - Symptom onset (COWS) began at mean of 15.8 hours with “gooseflesh” and ended at 29.3 hours with “restlessness”.  - Only the SOWS peak total score predicted taper completion.  - SOWS performed better than COWS in terms of etiology and sensitivity of opioid WS.  - Withdrawal in the clonidine and tramadol group occurred at 8 hours and lasted 13 hours before all symptoms occurred; withdrawal in buprenorphine group started at 5 hours with “feel like using” but no other symptoms were reported until 13 hours and all symptoms surfaced only after 34 hours. | 73.8 | NR |
| 8 | Firouzkouhi et al. (2016), Iran [33] | 40 opioid-dependent children (60% male, mean age 11.14 years) | Clonidine and hydroxyzine to manage opioid withdrawal, ibuprofen for musculoskeletal pain.  Comorbid physical and psychiatric disorders treated with additional pharmacotherapies as appropriate.  Psychoeducation to manage craving and anger. | - Length of stay.  - Duration and types of WS (documented by psychiatrist who conducted physical examinations). | - Mean length of stay was 10.8 days.  - Withdrawal symptoms lasted an average of 1.63 days.  - Musculoskeletal pain and diarrhoea were most common WS.  - Type and duration of WS did not differ across gender or age. | NR | NR |
| 9 | Garhy et al. (2019), Middle East [6] | 44 patients admitted for opioid detox (52 detox episodes) (100% male, mean age 26.7 years).  76.9% of episodes involved diagnosis of opioid dependence; 23.1% of episodes involved opioid dependence together with another drug. | Detox with loperamide and omeprazole | - Length of stay.  - Adverse events.  - Discharge AMA. | - Mean length of stay was 18.96 days; mean length of stay for incomplete and complete treatment was 4.62 days and 30.34 days respectively.  - No serious adverse events reported.  - 42.3% of detox episodes ended prematurely when the patient discharged themselves AMA. | 57.7 | NR |
| 10 | Guo et al. (2018), Singapore [74] | 111 patients with opioid dependence (92.8% male, mean age 44.1 years) | Randomised to either lofexidine or diazepam | - OOWS.  - Treatment retention rate.  - SOWS.  - VAS for opioid craving.  - Adverse events. | - OOWS, SOWS & VAS scores did not differ between groups.  - Change in pupil size lower in lofexidine group than diazepam group.  - Treatment retention higher in lofexidine group than diazepam; 25.5% of lofexidine group and 22.6% of diazepam group remained in the study at the end.  - No serious adverse events related to study medication reported. | 22.6-25.5 | NR |
| 11 | Hakansson & Hallén (2014), Sweden [91] | 122 patients with opioid dependence (81% male, mean age 34.3 years) | Buprenorphine detox | - Comparisons between patients who completed detox and those who dropped out | - 34% dropped out AMA.  - Mean length of stay for those discharged AMA and those who completed treatment was 6.7 days and 13.3 days respectively.  - Completers older and more likely to have a plan to enter inpatient or residential treatment after detox as opposed to outpatient treatment. | 66.0 | NR |
| 12 | Liebschutz et al. (2014), USA [34] | 145 opioid dependent patients (71.2% men, mean age 40.5 years) | Assigned to detox (n = 67) or linkage group (n = 72).  Detox group: Buprenorphine induction + 4 days of buprenorphine taper.  Linkage group: Buprenorphine induction, maintenance dose of buprenorphine during hospital stay and facilitated linkage to follow up care at hospital-associated OAT. | - Entry into hospital associated OAT program any time between intake and 6 months after intake.  - Length of illicit opioid use in the 30 days before 1-, 3- and 6-month interviews.  - Time to entry into OAT program.  - OAT days at 1, 3 and 6 months. | - Linkage group had higher follow-up rates at 6 months.  - 72.2% in the linkage group entered hospital’s OAT by 6 months vs. 11.9% from detox group.  - Linkage group had shorter time to OAT entry.  - 16.7% in linkage group were still in OAT at 6 months compared to 3.0% in detox group.  - Linkage group more likely to have abstained from illicit opioid use than detox group and had lower average days of illicit opioid use. | NR | NR |
| 13 | Liu et al. (2013), China [35] | 91 opioid-dependent patients (86.8% male, mean age 36.4) | 15-day program.  Methadone during days 1-3, then randomised to receive scopolamine or methadone. | - SOWS completed each day.  - Completion of inpatient detox.  - Heroin craving, depression, anxiety, measured using VAS, BDI and SAS at day 0 and end of inpatient detox.  - Working memory (digit span test) and d2 test at day 0 and end of detox. | - 46 participants (100%) in scopolamine group and 43 participants (95.5%) in the methadone group finished inpatient detox.  - SOWS in methadone group increased on days 11 and 12.  - SOWS higher in methadone than scopolamine group.  - No group differences in terms of VAS, BDI, SAS, working memory and d2 test. | 95.5-100.0 | NR |
| 14 | Mannelli et al. (2012), USA [36] | 127 opioid-dependent patients (66.1% male, mean age 32.2 years) | Detox using: NTX, NTX/CLO, CLO, or NTX-PLA | - Opioid withdrawal severity assessed using SOWS and OOWS.  - Retention in treatment.  - Use of ancillary medications. | - NTX had lower SOWS scores than CLO or NTX-PLA.  - NTX/CLO had attenuated withdrawal vs. NTX or CLO.  - OOWS scores lower in NTX than CLO or NTX-PLA- NTX/CLO had less shaking, anxiety, bone and muscle aches, restlessness, craving, lacrimation, rhinorrhoea and sweating than CLO.  - NTX had less muscle twitching, restlessness, anxiety and craving than CLO.  - 66.9% completed treatment; completion by group: 64.1% for NTX, 85.3% for NTX/CLO, 60.6% for CLO, 52.4% for NTX-PLA.  - No group differences in amount of ancillary medication.  - No serious adverse events. | 66.9 | NR |
| 15 | Mannelli et al. (2013), USA [37] | 174 opioid dependent patients (67.7% male, mean age 31.6 years) | 6-day program.  Methadone taper plus one of the following: NTX, NTX/CLO, CLO, or NONTX/CLO | - Opioid withdrawal (via SOWS) and tobacco craving (QSU-BREF) | - NTX associated with reduced withdrawal symptom severity.  - SOWS scores did not differ between groups.  - Patients who were also nicotine dependent and who were allowed to smoke during treatment reported higher opioid craving.  - Cigarette craving higher in patients who were nicotine dependent and who were allowed to smoke vs. those that were nicotine dependent but were not allowed to smoke.  - Smokers showed lower completion rates (no specific rate reported).  - Patients who were also nicotine dependent in the NTX group had lower SOWS scores than the CLO or NONTX/CLO group.  - Smokers in the NTX/CLO group had attenuated withdrawal versus CLO group.  - Opioid craving lower in smokers in the NTX group than CLO or NONTX/CLO group.  - Cigarette craving lower in NTX/CLO group than other groups.  - No group differences in use of ancillary medications. | 69.0 | NR |
| 16 | Naderi-Heiden et al. (2012), Austria [16] | 404 opioid-dependent inpatients (71.8% male, mean age 27.4 years) | Usually 2-4 week program using slow-release oral morphinsulphate/-hydrochloride, methadone or buprenorphine | - Number of deaths (observed during 11 years after completion) in detox completers vs. non-completers.  - Employment in the years after discharge. | - 35 died during the observation period, 21 of whom completed detox and 14 of whom did not.  - Age at detox initiation, completion of detox, gender and polysubstance dependence did not affect mortality.  - Pure opioid-dependence patients had higher employment rates following discharge than those with polysubstance-dependence. | 58.7 | NR |
| 17 | Pjrek et al. (2012), Austria [100] | 42 inpatients with opioid-dependence (61.9% male, mean age 29.4 years) | Detox with methadone or buprenorphine | - Self-reported SOWS for WS.  - Activity levels via actigraphy. | - Lower total and daytime activity in methadone group.  - Buprenorphine group had lower sleep efficiency, shorter sleep.  - No group differences on the SOWS. | NR | NR |
| 18 | Puffer et al. (2012), USA [81] | 45 opioid-dependent inpatients (68.9% male, age range from 18-57); mean age not reported | 3-4 day buprenorphine taper and ancillary medications as necessary, individual and group counselling, 12-step/mutual help programs, meetings with psychiatrist and case worker | - Assessed at baseline, 1 week and 2 weeks post discharge.  - Substance use/relapse via self-report and supervised urine samples.  - Self-reported religious coping via the Brief RCOPE.  - 12-step program participation (no. of days they had attended a 12-step meeting since previous follow-up). | - Following discharge, 47% of those who responded to follow-up had relapsed and were using opioids on average 15.9% of days during follow-up period.  - Frequency of opioid use not related to age, gender, education, years of substance use prior to admission, treatment condition.  - Majority Catholic (64%) and 25% had no religion.  - Change in religious coping from baseline to follow-up: 52% had increase in positive religious coping, 15% had decrease in positive religious coping, 33% had no change; 42% had decrease in negative religious coping, 27% had increase in negative religious coping, 30% had no change.  - Percentage of participants reporting no religious coping at all, decreased from baseline to follow up.  - Change in positive or negative religious coping not associated with religiosity, age, gender, education, or years of substance use.  - Positive religious coping associated with 12-step participation at follow-up.  - Change in positive religious coping not related to frequency of post-discharge opioid use.  - Reductions in negative religious coping related to lower post-discharge opioid use. | NR | NR |
| 19 | Sarkar et al. (2018), India [94] | 70 participants, 100% male:  30 inpatients with opioid dependence, mean age 31.0 years;  40 age-matched controls mean age 30.9 years. | 7-10 day detox via tapering doses of buprenorphine | - Serum BDNF taken on day 1 and 10 | - No change in BDNF levels.  - No group differences in BDNF levels between cases and controls. | NR | NR |
| 20 | Shulman et al. (2021), USA [92] | 283 opioid-dependent inpatients (68.8% male, mean age 34.0 years) | Varied detox methods:  3-7 methadone taper, 4–14-day buprenorphine taper, or clonidine and other non-opioid medications. | - Successful induction onto XR-NTX after detox | - 72.0% successfully inducted onto XR-NTX.  - Receiving methadone and buprenorphine during detox decreases chances of successful XR-NTX but this relationship is no longer significant when accounting for different detox sites.  - Differences in XR-NTX induction might be due to different medications or different sites. | NR | NR |
| 21 | Stein et al. (2020), USA [76] | 115 patients with OUD (68.2% male, mean age 32.4 years) | 5-day program.  Randomised to either:  WM group: Detox using buprenorphine/naloxone; LINK group: Detox using buprenorphine/naloxone and made a post-discharge appointment for the week after discharge. | - Assessed on day 5 (day of discharge), day 12 (1 week post discharge), 1 month, 3 months and 6 months after discharge.  - Illicit opioid use in the 30 days prior to the 1-, 3-, 6-month interviews.  - Self-reported prescribed buprenorphine and methadone use in the 30 days prior to 1-, 3-, 6-month interview. | - LINK group had lower chances of using more than 10 days in a month at day 12 but not 6 months, lower rates of illicit opioid use at all timepoints and, higher rates of frequent prescribed buprenorphine use (> 10 days) than WM.  - WM group had higher rates of prescription methadone use than LINK group. | NR | NR |
| 22 | Stewart et al. (2013), USA [55] | 95 pregnant opioid-dependent female inpatients, mean age 25.5 years | Opioid detox with methadone | - Successful detox (no maternal illicit drug supplementation at time of delivery).  - Duration of detox.  - Factors relating to success of detox. | - 56% had successful detox.  - Those who did not have successful detox were more likely to have positive hepatitis C antibody.  - No difference in HIV, syphilis, hepatitis B status, gestational age upon admission, maximum methadone dose required between successful and unsuccessful detox.  - Duration of detox longer in successful detox. | 56.0 | NR |

*Note.* AMA, against medical advice; ARSW, Adjective Rating Scale for Withdrawal; AS, Anxiety Severity; BDI, Beck Depression Inventory; BDNF, brain derived neurotrophic factor; Brief RCOPE, Brief Measure of Religious Coping; CLO, clonidine; COWS, Clinical Opiate Withdrawal Scale; ER, extended-release; LINK, linkage to office-based buprenorphine; NONTX/CLO, no naltrexone and no clonidine; NR, not reported; NTX, naltrexone; NTX-PLA, naltrexone placebo; OAT, opioid agonist treatment; OOWS, Objective Opiate Withdrawal Scale; OUD, opioid use disorder; QSU-BREF, Brief Questionnaire of Smoking Urges; SAS, Self-Rating Anxiety Scale; SOWS, Subject Opioid Withdrawal Scale; SUD, substance use disorder; VAS, Visual Analog Scale; WM, withdrawal management; WS, withdrawal severity; XR-NTX, Extended-Release Naltrexone.

Table 3. Summary of withdrawal treatment outcomes in inpatient settings for other drug users

|  | **Author (year), country [ref]** | **Sample** | **Program components** | **Outcome measures** | **Main findings** | **% Complete** | **% Transfer** | |
| --- | --- | --- | --- | --- | --- | --- | --- | --- |
| 1 | Allsop et al. (2015), Australia [70] | 38 cannabis-dependent patients (65.8% male, mean age 40.51 years) | 7-night program.  Randomised allocation to lithium carbonate 500 mg or placebo (lactose). | - Objective sleep patterns (actigraphy).  - CWS and subjective sleep patterns (self-report).  - Treatment retention. | - Subjects on lithium less fragmented sleep pattern than placebo.  - None of the objective measures of sleep quality predicted overall CWS nor any of the subjective withdrawal features in DSM-5.  - Sleep efficiency predicted treatment retention. | NR | NR | |
| 2 | Beaufort et al. (2017), The Netherlands [62] | 47 patients with SUD (substance type includes alcohol, cannabis, opiates, cocaine/crack, sedatives, GHB and stimulants); (77% male, mean age 43 years) | 10–15-day detox with medical and psychological support (no treatment for dependence or co-morbid disorders) | - DASS-21 measured at intake and 8-10 days after admission.  - MINI conducted 8-10 days after admission. | - Significant reduction in depression on DASS-21.  - Results from MINI interview showed that 24% currently had a depressive disorder.  - DASS-21 at intake lacks sensitivity and specificity in predicting depression; better results when using DASS-21 at 8-10 days after detox admission. | NR | NR | |
| 3 | Berman et al. (2019), Sweden [53] | 87 inpatients with SUD (75.9% male, mean age 37.2 years). | Randomised to either TAU or MI.  TAU comprised medication to manage withdrawal and referral to social or psychiatric services if necessary; no systematic counselling. | - MI adherence.  - Self-reported drug use (3 months post-discharge).  - Assessment of client and counsellor utterances during MI sessions. | - Client change talk is associated with motivation to change drug use behaviour.  - Counsellor change talk preceded client change talk. | NR | NR | |
| 4 | Bonnet et al. (2014), Germany [71] | 39 patients with cannabis dependence (79.5% male, mean age 28.6 years) | 16-day detox treatment comprising medical visits, movement therapy, occupational therapy, social counselling, one-to-one and group therapy involving motivational enhancement, cognitive-behavioural therapy, and psychoeducation, and referral to long-term rehabilitation | - Treatment completion.  - Mean treatment duration.  - Severity of WS: modified version of MWC used during interview and CGI-S.  - BPRS used to assess psychiatric burden at day 1 and end of study. | - Mean length of treatment was 18.5 days.  - Intensity of WS peaked on the fourth day; highest withdrawal severity was “moderate” in 7 patients, “marked” in 16 patients and “severe” in 16 patients.  - Intensity of individual WS peaked at day 4 except for craving and sleeplessness, which peaked on day 2. All symptoms were mild to moderate in severity.  - Women showed more severe WS than men.  - Negative association between withdrawal symptom severity and serum levels of THC, THC-OH and THC-COOH at baseline. | 87.1 | NR | |
| 5 | Bonnet et al. (2015), Germany [54] | 35 patients with cannabis dependence (80% male, mean age not reported) | 16-day program comprising medical visits, movement therapy, occupational therapy, social counselling, one-to-one and group therapy involving motivational enhancement, cognitive-behavioural therapy, and psychoeducation, and referral to long-term rehabilitation | - Treatment completion.  - Length of treatment.  - Course of psychiatric comorbidity and psychiatric symptoms: HAMD, HAMA, YMRS, BPRS and SCL-90-R.  - Relationship between psychiatric scales and serum levels of THC and its metabolites | - Median length of treatment was 16.8 days.  - Patients with psychiatric comorbidity remained stable during detox (no additional treatment needed during the study); all psychiatric symptoms (mean SCL-90-R) decreased during detox.  - No relationship between serum THC and psychiatric symptoms beyond a positive association between serum THC and cognitive impairment. | 88.6 | NR | |
| 6 | Claus et al. (2020), Germany [82] | 78 adult inpatients with cannabis dependence (77% male, mean age 26.4 years) | Up to 24 days of treatment.  Medical assessments, individual and group therapy sessions involving motivational enhancement, cognitive-behavioural interventions and social counselling. All patients offered transfer to long-term rehabilitation after discharge. | - Treatment duration and attrition.  - Withdrawal symptom severity measured via modified MWC as a face-to-face interview and the CGI-S.  - Urine cannabinoids. | - 11.5% dropped out of treatment.  - Mean length of treatment was 14.6 days.  - WS severity positively correlated with THC-COOH in patients with severe WS only.  - Urine THC-COOH decreased more quickly in males than females. | 88.5 | NR | |
| 7 | Corominas-Roso et al. (2015), Spain [83] | 40 inpatients with cocaine dependence (95% male, median age 32.5 years) | 12-day program with 22 weeks follow-up.  Two groups: caffeine and biperiden versus placebo.  Lorazepam given for treatment of anxiety and insomnia. | - Relapse.  - Serum BDNF levels, which are involved in cocaine reward and consumption (i.e., increases with cocaine intake). | - 20 relapsed within 14 days after discharge (early relapsers), 18 relapsed between 14 and 90 days post-discharge (late relapsers), 2 remained abstinent past the follow-up period.  - No differences between caffeine and placebo group in terms of BDNF serum levels, both at baseline and post-detox.  - BDNF serum levels unchanged in early relapsers but increased in late relapsers; baseline serum BDNF levels higher in early relapsers than late relapsers. | NR | NR |  |
| 8 | Corominas-Roso et al. (2013), Spain [58] | 40 cocaine-dependent patients (92.5% male, mean age 33.9 years).  46 healthy controls (matched for age and gender; 89.1% male, mean age 35.5 years). | 12-day program.  Caffeine and biperiden versus placebo.  No other medication given except lorazepam for insomnia. | - Serum BDNF | - Serum BDNF correlated with baseline depression and abstinence in patients who have never experienced psychotic symptoms under the influence of cocaine, but no correlation in patients with a history of psychotic symptoms under the influence of cocaine.  - Change in serum BDNF levels predicted baseline depression but not abstinence. | NR | NR |  |
| 9 | de Haan et al. (2014), The Netherlands [56] | 130 inpatients with AOD (77.7% male, mean age 39.3 years) | Maximum 21 days.  Benzodiazepines and/or methadone used for withdrawal management; program did not include a psycho-therapeutic intervention | - Dropout rate.  - Measurement of alexithymia via TAS-20 (3 factors: DIF, DDF, EOT).  - Subjective Withdrawal Symptoms. | - 30 of the 130 patients dropped out before follow-up  - At follow up (maximum 3 weeks from baseline), total TAS-20, DDF, EOT scores increased for low-alexithymia patients; DIF scores decreased in moderate-alexithymia patients; all TAS-20 variables decreased for high-alexithymia patients  - WS decreased from baseline to follow up | NR | NR |  |
| 10 | Johnston et al. (2014), Australia [38] | 38 patients with cannabis dependence (65.8% male, mean age 40.51 years) | Lithium carbonate or placebo during 8-day detox | - Blood samples taken on days 1, 2, 4, 7.  - Follow up interviews at 14, 30, 90 days after discharge (self-reported recent cannabis and other substance use, Cannabis Problems Questionnaire, Short Form-12, the WHOQOL– BREF and DASS–21).  - CWS measured once daily.  - Detox completion.  - Safety and tolerability assessed daily by nursing staff using lithium adverse event checklist; self-reported trial medication satisfaction ratings. | - Mean no. of days in treatment was 5.18 and 5.94 for placebo vs. lithium groups respectively.  - 50% of lithium group completed detox vs. 41% of the placebo group.  - Total CWS scores decreased over the 7 days of detox; no main effect of lithium treatment, no time x treatment interaction.  - No difference in adverse effects between groups.  - Plasma oxytocin, THC-COOH and THC levels decreased over time but no difference between groups.  - Improvements in all interview measures between baseline and follow-up, but no between-group differences except for the WHOQOL-BREF physical health scores. | 41.0-50.0 | NR |  |
| 11 | Kamal et al. (2016), Netherlands [25] | 229 patients with GHB misuse (100% female, mean age 29.1 years) | Management of WS via GHB, doses according to symptoms (self-reported and nurse/doctor’s observations) | - Subjective WS via SOWS within 5 hours after cessation of illicit GHB | - Participants divided into 3 groups according to their drug use: GO, GSE, or GST.  - GO and GSE groups showed stable general withdrawal symptom intensity.  - GST groups showed decrease in severity of total WS. | NR | NR |  |
| 12 | Kawasaki et al. (2012), USA [24] | 310 inpatient admissions with benzodiazepine dependence (55% male, median age 36 years) | Phenobarbital taper, 3-day schedule | - Adverse events (seizures, falls, delirium).  - Re-admitted to hospital or emergency room visit within 30 days of discharge.  - Doses held because of sedation.  - Completion rate. | - No seizures, only 3 (1%) had delirium.  - 7.1% left treatment early AMA.  - 7.1% visited the emergency department within 30 days of discharge.  - 6.1% were re-admitted to the hospital’s medical or psychiatry service, but only 3 of these were admissions for benzodiazepine withdrawal.  - At least one dose of the taper was held because of sedation in 25.8% of patients. | 92.9 | NR |  |
| 13 | Kiepek et al. (2015), Canada [30] | 109 mixed AOD (112 admissions) (33.9% male, age range 18-70 years); mean age not reported | Buprenorphine-naloxone taper.  Successful completion defined as 7 days of admission or completion of withdrawal management in addition to 3 days without medications for withdrawal management. | - Length of stay.  - Successful completion of detox.  - Substance use after discharge (2 weeks, 3 months, 6 months). | - Length of stay ranged from 1-29 days, mean 12 days.  - 81% successfully completed detox.  - 46%, 32% and 30% of patients who were admitted for opiate use (*n* = 91) reported opiate use at 2 weeks, 3 months and 6 months respectively.  - 10% and 5% reported lapse in opiate use (used only once or twice) at 2 weeks and 3 months.  - 9%, 10%, 3% reported relapse (using at least 25% intake dose and frequency) at 2 weeks, 3 months and 6 months. | 81.0 | NR |  |
| 14 | Lee at al. (2014), USA [72] | 72205 inpatients mixed AOD (63% male) from different facilities.  Ages reported in categories ranging from 18 to 45+. | Medically managed withdrawal treatment with varying levels of intensity across 5 states in the USA | - Impact of continuity of care (continuing treatment within 14 days of discharge) on readmission to detox | - Continuity of care rates ranged from 12.5 to 45.5% across different facilities.  - Clients with no continuity of care are more likely to have a readmission detox episode that is not followed up by treatment. | NR | 12.5-45.5 |  |
| 15 | Lennox & Cecchini-Sternquist (2018), USA [48] | 107 mixed AOD (67.9% male, mean age 28.4 years) | Medical oversight provided.  Program comprised 20-30 minutes of moderate aerobic exercise, gradually increasing doses of crystalline niacin, nutritional supplementation, moderate-temperature sauna therapy for about 4.5 hours a day.  Program typically completed in 2-4 weeks. | - SF-36: health-related quality of life.  - Treatment Process Questionnaire: client’s understanding, satisfaction and perceived benefit of treatment.  - ASI and GAIN-SS measured at intake and discharge, followed up at 6 months and 1 year.  - Treatment length and completion rate. | - Mean length of treatment was 102 days; time spent in the detox itself was about 29 days, with 2 weeks spent in treatment before starting detox.  - ASI scores decreased from baseline to discharge.  - GAINS-SS scores decreased from baseline to discharge when assessing past 30 days and 90 days but not past year. | 99.0 | NR |  |
| 16 | Levandowski et al. (2016), Brazil [75] | 108 female patients with crack cocaine use disorder (mean age 29.0) and 24 healthy female control participants (mean age 31.5) | 21 days of inpatient treatment.  No access to alcohol, cigarettes, or drugs; daily psychoeducation, relapse prevention, support groups; access to first generation antipsychotics for psychomotor agitation, or mood stabilisers and antidepressants if necessary; constant nursery care, occupational therapy, nutritional care, light physical activity thrice a week. | - Severity of crack cocaine WS on day 4, 11, 18 measured using the CSSA.  - Severity of depressive symptoms using BDI-II on day 4, 11, 18.  - Blood samples collected on day 4, 11, 18. | - Patients with childhood maltreatment had higher BDI-II scores and worse WS.  - Regardless of childhood maltreatment history, all Th1 and Th17-related cytokine levels decrease in week 1 of detox relative to controls, then these levels approach control values after 3 weeks of treatment. | NR | NR |  |
| 17 | Li et al. (2013), Canada [42] | 2231 unique clients mixed AOD (64.7% male, mean age 40.5 years) | 24-hour nursing staff, onsite medical care, medical management of WS and other health concerns.  Included individual counselling, educational groups, 12-step programs, acupuncture, other alternative therapies. | - Discharged AMA rate (initiated treatment, did not complete).  - Predictors of leaving AMA. | - Discharged AMA rate was 21.1% (n = 309, of which 69 were Aboriginal).  - For Aboriginal group, being female or having a hepatitis C infection were more likely to drop out of treatment.  - For non-Aboriginal group, older clients and clients whose primary substance of abuse was opiates were more likely to drop out of treatment. | 78.9 | NR |  |
| 18 | Lin et al. (2014), Taiwan [97] | 80 heroin-dependent patients, 15 discharged AMA within 2 days, so final sample was 65 (96.9% male, mean age 35.7 years) | Clonidine detox, decreasing dosage over 7 days.  Participants randomised to receive dextromethorphan or placebo. | - Severity of heroin WS measured using OOWS measured 3 times a day every day.  - Total sleep time.  - CGI-S indicating drug efficacy at discharge.  - Patient’s treatment satisfaction.  - Total amount of ancillary medications. | - WS increased in severity from day 1 to 2 then improved slowly.  - Dextromethorphan group had less severe WS from day 3 to day 6.  - Joint and muscle aches significantly better on days 4 and 5 in the dextromethorphan group vs. placebo.  - No group differences in no. of sleep hours, CGI-S, satisfaction scores, or total amount of ancillary medications used. | NR | NR |  |
| 19 | Manning et al. (2019), Australia [14] | 47 methamphetamine-dependent inpatients (53% male, mean age 34.67 years) | 7-10 days on average (range from 3-15 days in this sample).  Medical management of WS and group therapy.  Attentional bias modification intervention using methamphetamine-related imagery. | - Substance use in past 2 weeks at 2-week follow up and in past 30 days before 3-month follow up (self-report).  - Acceptability of intervention (self-report).  - Completion rates.  - Craving (MCQ). | - In the 29 participants who completed the MCQ both before and after intervention, MCQ scores decreased from session 1 to session 4 (last session).  - Of 31 who completed the 2-week follow-up, 61% remained abstinent; half of those who relapsed only used methamphetamine on 1 day in the 2 weeks before the follow-up.  - Of 26 who could be contacted at 3-month follow-up, 54% remained abstinent; the 12 who reported using methamphetamine averaged 12.1 days of use in the past 30 days. | 62.0 | NR |  |
| 20 | Marhe et al. (2013), The Netherlands [47] | 68 heroin-dependent inpatients (85.3% male, mean age 40.9 years).  Most participants were also cocaine dependent (88.1%). | 3-week program aimed at reducing physical and mental heroin WS.  Placed on methadone maintenance at admission. | - Relapse from day 3 to day 9 of treatment (i.e., early relapse).  - Relapse after study week but during detox (i.e., late relapse).  - Self-reported craving for heroin and cocaine on an ecological momentary assessment.  - Self-reported attitude to heroin and cocaine on an ecological momentary assessment.  - Drug Stroop effect.  - Drug (IAT) effect.  - Number of temptation episodes (i.e., when participants felt like using heroin or cocaine). | - Both early and late relapsers did not have more temptation episodes than those who did not relapse (either at all or during the study).  - Early relapsers had greater attentional bias and more positive implicit attitudes than those who did not relapse during the study during temptation episodes.  - Compared to those who did not relapse during the study, early relapsers had greater craving and more positive explicit attitudes towards drugs during temptation episodes compared to random assessments (craving/attitudes tested during non-temptation episodes).  - Late relapsers had more positive explicit attitudes towards drugs at temptation assessments than at random assessments compared to those who never relapsed.  - Increased attentional biased during temptation episodes predicted relapse. | NR | NR |  |
| 21 | Mash et al. (2018), USA [64] | 89 cocaine (66.7% male, mean age 36.1 years) and 102 opioid dependent (66.7% male, mean age 35.8 years) inpatients | 5-day detox with ibogaine hydrochloride (HCl) | - Self reported craving for cocaine or opioids using HCQ-29 and CCQ-29.  - OOWS assessed by physicians.  - BDI-II, POMS second edition, SCL-90 for depressive symptoms.  - Side effects of ibogaine (i.e., safety) | - Ibogaine was well tolerated; nausea/vomiting and ataxia of gait most common side effects.  - Acute WS begin about 8 hours after last heroin dose, peaks at 1-2 days and resolves around 7-10 days; significant reduction in self-reported WS 72 hours after recovery from ibogaine treatment compared to pre-ibogaine levels.  - Opioid-dependent and cocaine-dependent participants had reduced drug craving on HCQ-29 and CCQ-29 respectively at both post-treatment and at 1-month follow-up compared to baseline.  - BDI-II score decreased at 1 month follow up compared to measurements at baseline and program discharge.  - Improvement in mood scores for all 3 mood assessments for opioid-dependent participants.  - Improved mood scores for all 3 mood assessments for cocaine-dependent participants, who scored lower on all 3 assessments at discharge and 1 month follow up compared to opioid-dependent participants. | NR | NR |  |
| 22 | McKetin et al. (2012), Australia [63] | 112 mixed AOD with majority having methamphetamine dependence.  72% male, mean age 31.7 years. | About 1 week on average, medical management of WS | - Self-reported methamphetamine use over a 3-year follow-up period (measured at 3 months, 1 year, 3 years) | - Methamphetamine use reduced over follow-up period but frequency of methamphetamine use did not differ from controls at any measurement point (3 months, 1 year, 3 years).  - Detox alone does not change methamphetamine use at any follow-up compared to no treatment | NR | NR |  |
| 23 | Mills et al. (2014), Australia [77] | 29 mixed AOD (46.4% male, mean age 37.9 years) | Medicated detox (mean 7.12 days)  Study included a brief intervention comprising a one-hour session on psychoeducation regarding the relation between trauma and substance use and symptom management | - Client satisfaction at 1 week follow up (self-reported via CSQ).  - The following measurements were taken at 1 week, 1 month and 3 months after the brief intervention:  - PTSD symptom severity (Clinician Administered PTSD Scale) and post-traumatic cognitions (Post Traumatic Cognitions Inventory);  - Lifetime and current substance use (via Opiate Treatment Index);  - Severity of dependence (via the Severity of Dependence Scale). | - PTSD symptom severity decreased from baseline to 1-week follow-up and from 1-week to 3-month follow up; significant reductions in avoidance/numbing symptoms, hyperarousal symptoms (which decreased from baseline to 1-month follow-up and then remained stable) and re-experiencing symptoms (only at 3-month follow-up).  - No change in negative post-traumatic cognitions.  - Majority continued to meet criteria for PTSD.  - Use of main drug of concern decreased from baseline to 1-week follow-up, remained stable through to 1-month follow-up and thereafter increased again, but this final rate of use was still lower than baseline.  - Same pattern observed in terms of dependence.  - High level of satisfaction with outcome. | NR | NR |  |
| 24 | Mongeau-Pérusse (2021), Canada [49] | 78 cocaine dependent inpatients (82.1% male, mean age 45.9) | 10-day inpatient detox, including medical care and psychoeducation group therapy.  Detox using CBD vs. placebo. | - Drug cue-induced craving on day 8.  - Stress-induced craving (difference in craving scores [measured via VAS] before and after a stress-induced craving session on day 8).  - Adverse events and serious adverse events assessed via the Systematic Assessment for Treatment Emergent Events.  - Completion of detox.  - Time to relapse within 12 weeks (self-report and urinalysis). | - No significant change from baseline craving scores in response to drug- or stress-induced craving.  - 42.5% of those in the CBD group reported at least one adverse event, most commonly diarrhoea and nausea.  - No evidence for CBD being more effective than placebo in treatment of cocaine dependence.  - Risk of cocaine relapse similar for both groups. | 79.5 | NR |  |
| 25 | Pérez de los Cobos et al. (2021), Spain [79] | 87 cocaine-dependent inpatients (68.0% male, mean age 39.5 years) | 14 days on average.  Pharmacological management of WS using benzodiazepines and/or antipsychotics. | - Cocaine craving assessed by CSSA administered by nurses.  - Anxiety and depression measured using STAI and BDI respectively. | - Cravers had longer admissions than non-cravers.  - Absence of craving occurred with lower anxiety and depression.  - Lower cocaine use 30 days before admission predicts craving absence. | NR | NR |  |
| 26 | Person et al. (2021), USA [57] | 832 mixed AOD who left detox AMA (69.7% male, mean age 35.2 years) | Methadone for opiate withdrawal or chlordiazepoxide or lorazepam for alcohol or benzodiazepine withdrawal | - Factors associated with leaving AMA | - Increased age, detox using a lorazepam protocol and personal obligation as reasons for discharge AMA correlated with increased length of stay in hospital before discharging AMA.  - Substance use, medical history, psychiatric history did not correlate with discharge AMA.  - Days 2 to 4 were peak periods for discharge AMA. | NR | NR |  |
| 27  *(Continues)* | Silverman (2016), USA [60] | 144 substance-dependent inpatients (substance type includes alcohol, cocaine/crack, heroin, prescription drugs and others); (54.9% male, mean age 36.8 years) | Typically 3-5 days.  Medical detox, minimal psychosocial treatment.  Experimental group also participated in music therapy lyric analysis sessions to distract patients from craving and withdrawal. | - Self-reported WS and craving via ARSW | - No difference in withdrawal or craving between experimental group and control (only a numerically different result in favour of experimental group) | NR | NR |  |
| 28 | Sofin et al. (2017), Germany [9] | 832 mixed AOD (74% male, mean age 44 years) | 12-16 days on average.  Clomethiazole for alcohol detox, methadone for opioid detox, abrupt cessation with medical support for cannabis, amphetamines and cocaine detox.  Group therapy, psychoeducation, attendance of 5 self-help groups outside the clinic in preparation for transition into long-term care. | - Premature treatment dropout.  - Predictors of dropout. | - Those who dropped out were usually younger and more likely to be male.  - Being female, living in a partnership, had children and were employed, were well-educated and spoke German as a native language were more likely to complete treatment.  - Patients with no previous dropouts more likely to complete treatment.  - Longest period of abstinence not predictive of drop out. | 63.1 | NR |  |
| 29 | Stevens et al. (2015a), Belgium [78] | 70 mixed AOD (90% male, mean age 28.4 years) | Medical management of WS, crisis support, enhancement of motivation for abstinence, information and advice for further treatment, referral to long-term treatment | - Short-term relapse (3 months after discharge) according to Interview for IRAB | - Relapsers show greater devaluation of delayed rewards on delay discounting task and are more guided by immediate gratification rather than long-term rewards on the Iowa Gambling Task (i.e., ability to delay gratification was taken to be a characteristic or trait possessed by a person that would affect relapse; delay discounting was not measured as an outcome measure).  - Delay discounting and impulsive decision-making independently predict relapse. | NR | NR |  |
| 30 | Stevens et al. (2015b), Belgium [28] | 84 mixed AOD (87% male, mean age 28.71 years) | Typically 5-6 weeks.  Medical management of WS, crisis support, enhancement of motivation for abstinence, information and advice for further treatment, referral to long-term treatment. | - Treatment retention | - Pre-treatment delay discounting predicts treatment retention; greater delay discounting predicts shorter treatment retention and higher likelihood of premature dropout | 44.0 | NR |  |
| 31 | Timko et al. (2019), USA [73] | 298 alcohol and/or opioid dependent inpatients, randomised into intervention or TAU.  148 in intervention group, 95.3% male, mean age 51.4 years.  150 in TAU group, 94.7% male, mean age 48.9 years. | TAU: Medically supervised withdrawal.  Intervention: TAU plus one 50-min individual session during inpatient stay and 12 weekly phone calls to enhance motivation for abstinence | - ASI measuring psychiatric symptoms, alcohol and drug use severity, and no. of days of alcohol and opioid use in the past 30 days assessed 3 months and 6 months after detox.  - Self efficacy at 3 and 6 months after detox measured with Brief Situational Confidence Questionnaire.  - Brief Addiction Monitor to measure substance use and risk factors for substance use.  - Re-entry into inpatient detox.  - 12-step group attendance and outpatient treatment attendance at 3 and 6 months. | - Intervention group less likely to receive repeated inpatient detox, less severe psychiatric symptoms, less substance use.  - Both groups equally likely to attend outpatient follow-up care or mutual-help groups.  - TAU group more likely to have attended 12-step group meetings at 6-month follow up; no group differences at 3-month follow up.  - No group differences for outpatient treatment attendance. | NR | 17.9-34.7 |  |
| 32 | Vederhus et al. (2015), Norway [65] | 140 SUD patients: 68 in motivational interviewing group, 72% male, mean age 43 years; 72 in brief advice group, 63% male, mean age 40 years. | Brief advice group: Medically supervised withdrawal, briefly advised to attend 12-step meetings and given brochure.  Motivational interviewing group: Same as Brief advice with motivational intervention consisting of 2 weekly 30-miniute educational and motivational sessions. | - Measures taken at 6-month follow-up.  - 12-step group affiliation measured with AAAS: Meeting attendance and 12-step group involvement.  - Substance use & SUD severity measured with semi-structured EuropASI. | - AAAS at 6-month follow-up was higher for Motivational interviewing group than Brief advice group.  - Motivational interviewing group attended 2x more meetings than Brief advice group but this difference was not statistically significant.  - Motivational interviewing group had fewer days of alcohol and drug use.  - Problem severity on ASI did not differ between groups.  - Abstinence was higher in those who attended 12-step meetings than those who did not. | NR | NR |  |
| 33 | Viola et al. (2014a), Brazil [50] | 104 female crack cocaine dependent inpatients (mean age 28.9 years).  20 female controls (mean age 29.5 years). | Usually, 3 weeks of treatment.  Symptomatic cocaine detox protocol including neuroleptics, antidepressants, mood stabilisers. | - Blood samples to assess plasma NF levels | - GDNF, NGF, NT-3 and NT4/5 levels lower in patients than controls.  - BDNF higher in patients than controls.  - GDNF plasma levels in patients with a history of childhood sexual abuse rose sharpy during 3 weeks of detox, while those with no history of childhood sexual abuse had lower and more stable GDNF levels during the same period. | NR | NR |  |
| 34 | Viola et al. (2014b), Brazil [99] | 93 female cocaine-dependent inpatients (mean age 28.4) | 3 weeks of drug rehabilitation, psychoeducation, support groups, nursing care, moderate amounts of physical activity, balanced diet, medical treatment | - Cocaine WS measured on day 4, 9, 14 using CSSA.  - 2.5 year follow up of number of rehospitalisations for cocaine dependence. | - Using cannabis from a young age and long-term cannabis use correlated with more severe cocaine WS and craving.  - Long-term cannabis use predicted more rehospitalisations 2.5 years after first detox. | NR | NR |  |
| 35 | Wechsberg et al. (2012), Russia [51] | 100 female heroin-dependent patients (mean age 25.9 years) | Usually 3-4 weeks of treatment.  Clonidine, non-opioid analgesics, hypnotics, antidiarrheal used to treat heroin WS; individual and group cognitive and behavioural relapse prevention therapy; no use of opioid agonist medications.  Participants randomised into 2 conditions on top of regular detox:  Nutrition intervention vs. Woman-Focused intervention that empowers women through educational activities, skill-building demonstrations, guided practice, role-playing. | - HIV risk behaviours among injecting drug users and drug use measured using the Revised Risk Behaviour Assessment | - Participants in both groups reduced drug injection frequency after detox.  - Woman-focused condition had more improvements in sex-risk behaviours. | NR | NR |  |
| 36 | Zaparte et al. (2015), Brazil [93] | 30 female crack-cocaine patients (mean age 29.17 years).  30 female healthy controls (mean age 29.56 years). | 21-day program.  No access to drugs, alcohol, cigarettes; no benzodiazepines prescribed during detox. | - Assessed on day 4 and 18 of detox.  - Blood samples (Blood protein content, protein thiol content, protein carbonylation, reduced glutathione, total reactive antioxidant potential).  - CSSA and BDI. | - Patients had decreased withdrawal and depressive symptoms on the CSSA and BDI respectively.  - At detox start, patients had more protein modification than controls (differences in protein carbonyl and thiol content).  - After 14 days of detox, carbonyl levels were similar in both groups (decreased protein carbonylation in acute abstinence).  - Drug abstinence associated with increase in total reactive antioxidant potential antioxidant capacity.  - Intensified antioxidant defences at end of detox.  - Superoxide dismutase levels inversely correlated with depressive symptom severity, withdrawal severity and ASI drug dependence severity.  - Protein thiol content at start and end of detox positively correlated with depressive symptom severity.  - Protein carbonyl levels positively correlated with ASI drug dependence severity. | NR | NR |  |

*Note.* Berman et al., (2019) and Vederhus et al., (2015) did not specify the type of substance used by their target population. AAAS, Alcoholics Anonymous Affiliation Scale; AMA, against medical advice; AOD, alcohol and other drugs; ARSW, Adjective Rating Scale for Withdrawal; ASI, Addiction Severity Index; BDI, Beck Depression Inventory First Edition; BDI-II, Beck Depression Inventory Second Edition; BDNF, Brain Derived Neurotrophic Factor; BPRS, Brief Psychiatric Rating Scale; CBD, cannabinol; CCQ-29, Cocaine Craving Questionnaire; CGI-S, Clinical Global Impression Scale – Severity of Illness; CSQ, Client Satisfaction Questionnaire; CSSA, Cocaine Selective Severity Assessment; CWS, Cannabis Withdrawal Scale; DASS-21, Depression, Anxiety and Stress Scale – 21 Items; DDF, Difficulty Describing Feelings; DIF, Difficulty Identifying Feelings; EOT, externally oriented thinking; EuropASI, European version of ASI; GAIN-SS, Global Appraisal of Individual Needs - Short Form; GDNF, Glial Cell-Line Derived Neurotrophic Factor; GHB, gamma hydroxybutyrate; GO, GHB-dependent without current co-use of other substances; GSE, GHB-dependent with current alcohol and/or sedative co-use; GST, GHB-dependent with current stimulant co-use; HAMA, Hamilton Anxiety Rating Scale; HAMD, Hamilton Depression Rating Scale; HCQ-29, Heroin Craving Questionnaire; IAT, Implicit Association Test; IRAB, Interview for Research on Addictive Behaviour; MCQ, Methamphetamine Craving Questionnaire; MI, motivational interviewing; MINI, Mini International Neuropsychiatric Interview; MWC, Marijuana Withdrawal Checklist; NF, neurotrophic factor; NR, not reported; NT, neurotrophin; OOWS, Objective Opiate Withdrawal Scale; POMS, Profile Of Mood States; PTSD, post-traumatic stress disorder; SCL-90-R, Symptom Checklist-90-Revised; SF-36, RAND Medical Outcomes Study Short Form Health Survey; SOWS, Subject Opioid Withdrawal Scale; STAI, State-Trait Anxiety Inventory; SUD: substance use disorder; TAS-20, Toronto Alexithymia Scale; TAU, treatment as usual; THC, tetrahydrocannabinol; THC-COOH, 11-Nor-9-carboxy-delta-9-tetrahydrocannabinol; THC-OH, 11-hydroxy-THC; VAS, Visual Analog Scale; WHOQOL-BREF, Abbreviated World Health Organization Quality For Life Scale; WS, withdrawal severity; YMRS, Young Mania Rating Scale.
